# MR-PheWAS: exploring the causal effect of SUA level on multiple disease outcomes by using genetic instruments in UK Biobank

**DOI:** 10.1136/annrheumdis-2017-212534

**Published:** 2018-02-06

**Authors:** Xue Li, Xiangrui Meng, Athina Spiliopoulou, Maria Timofeeva, Wei-Qi Wei, Aliya Gifford, Xia Shen, Yazhou He, Tim Varley, Paul McKeigue, Ioanna Tzoulaki, Alan F Wright, Peter Joshi, Joshua C Denny, Harry Campbell, Evropi Theodoratou

**Affiliations:** 1 Centre for Global Health Research, Usher Institute of Population Health Sciences and Informatics, University of Edinburgh, Edinburgh, UK; 2 Centre for Population Health Sciences, Usher Institute of Population Health Sciences and Informatics, University of Edinburgh, Edinburgh, UK; 3 Colon Cancer Genetics Group, Medical Research Council Human Genetics Unit, Medical Research Council, Institute of Genetics and Molecular Medicine, University of Edinburgh, Edinburgh, UK; 4 Department of Biomedical Informatics, Vanderbilt University Medical Center, Nashville, Tennessee, USA; 5 Department of Medical Epidemiology and Biostatistics, Karolinska Institutet, Stockholm, Sweden; 6 West China School of Medicine, West China Hospital, Sichuan University, Sichuan, China; 7 Public Health and Intelligence, NHS National Services Scotland, Edinburgh, UK; 8 Department Epidemiology and Biostatistics, School of Public Health, Imperial College London, London, UK; 9 MRC-PHE Centre for Environment, School of Public Health, Imperial College London, London, UK; 10 Department of Hygiene and Epidemiology, University of Ioannina Medical School, Ioannina, Greece; 11 Medical Research Council Institute of Genetics and Molecular Medicine, University of Edinburgh, Edinburgh, UK; 12 Edinburgh Cancer Research Centre, Institute of Genetics and Molecular Medicine, University of Edinburgh, Edinburgh, UK

**Keywords:** epidemiology, gene polymorphism, gout

## Abstract

**Objectives:**

We aimed to investigate the role of serum uric acid (SUA) level in a broad spectrum of disease outcomes using data for 120 091 individuals from UK Biobank.

**Methods:**

We performed a phenome-wide association study (PheWAS) to identify disease outcomes associated with SUA genetic risk loci. We then implemented conventional Mendelianrandomisation (MR) analysis to investigate the causal relevance between SUA level and disease outcomes identified from PheWAS. We next applied MR Egger analysis to detect and account for potential pleiotropy, which conventional MR analysis might mistake for causality, and used the HEIDI (heterogeneity in dependent instruments) test to remove cross-phenotype associations that were likely due to genetic linkage.

**Results:**

Our PheWAS identified 25 disease groups/outcomes associated with SUA genetic risk loci after multiple testing correction (P<8.57e-05). Our conventional MR analysis implicated a causal role of SUA level in three disease groups: inflammatory polyarthropathies (OR=1.22, 95% CI 1.11 to 1.34), hypertensive disease (OR=1.08, 95% CI 1.03 to 1.14) and disorders of metabolism (OR=1.07, 95% CI 1.01 to 1.14); and four disease outcomes: gout (OR=4.88, 95% CI 3.91 to 6.09), essential hypertension (OR=1.08, 95% CI 1.03 to 1.14), myocardial infarction (OR=1.16, 95% CI 1.03 to 1.30) and coeliac disease (OR=1.41, 95% CI 1.05 to 1.89). After balancing pleiotropic effects in MR Egger analysis, only gout and its encompassing disease group of inflammatory polyarthropathies were considered to be causally associated with SUA level. Our analysis highlighted a locus (*ATXN2/S2HB3*) that may influence SUA level and multiple cardiovascular and autoimmune diseases via pleiotropy.

**Conclusions:**

Elevated SUA level is convincing to cause gout and inflammatory polyarthropathies, and might act as a marker for the wider range of diseases with which it associates. Our findings support further investigation on the clinical relevance of SUA level with cardiovascular, metabolic, autoimmune and respiratory diseases.

## Introduction

Uric acid (UA) is the end product of the exogenous and endogenous purine metabolism, catalysed by the action of xanthine oxidase.^1^ Due to the evolved loss of uricase enzyme, humans are unable to convert UA into highly soluble compounds, leaving urate circulating in the blood and resulting in a high basal level of serum uric acid (SUA).^2^ The prevalence rate of hyperuricaemia (elevated SUA level >7.0 mg/dL) is in the range of 5%–25% across different countries.^3–5^ A progressively rising trend of hyperuricaemia prevalence has been observed worldwide.^5^ Concernedly, hyperuricaemia is thought to inflict multiple clinical consequences, which is believed to be causally related to gout and suggestively associated with a number of prevalent health conditions, such as cardiovascular and metabolic diseases.^6–8^

Our recently published umbrella review presented a comprehensive overview of the breadth of disease outcomes related to SUA level by incorporating evidence from multiple sources.^9^ A large number of disease outcomes were reported to be associated with SUA level in observational studies, covering a wide range of diseases, including cardiovascular disease, metabolic syndrome, diabetes, cancer and neurological disorders. However, evidence as to whether these associations are actually causal is not yet well developed, given that observational associations are susceptible to a variety of biases, confounding and/or reverse causality. Although results from randomised controlled trials (RCTs) have provided some evidence about the beneficial effects of SUA-lowering therapy on some intermediate traits or biomarkers (eg, blood pressure, endothelial function, serum creatinine), there remains a lack of RCTs focusing on the more important clinical disease endpoints.^10–12^ A number of Mendelian randomisation (MR) studies, using the genetic variants influencing SUA level as instruments, provide alternative evidence to distinguish causal from non-causal associations. However, these MR studies examined a limited set of disease outcomes and were not able to detect moderate effect size due to limited power.^13–19^ Increasing sample size and the range of outcomes in an enlarged MR study thus offers the prospect of deeper and wider insight into the causal role of SUA.

MR analysis is typically hypothesis-driven based on prior knowledge to specify the outcome to be examined in relation to the exposure of interest. Traditionally, only one (or a limited number) association between the exposure and one (or a few) predefined outcome(s) is tested in an MR study. Recently, phenome-wide Mendelian randomisation (MR-PheWAS) analysis has been proposed by integrating the phenome-wide association study (PheWAS) and MR method to build a hypothesis-searching approach, which aims to explore potential causal relationships between an exposure (using genetic instruments as proxies) and a range of phenome-wide disease outcomes in a high-throughput manner.^20^ This approach is effective in evaluating or replicating the associations reported in observational studies, as well as discovering new relationships and generating new hypotheses on the genetic architecture shared by the related phenotypes. With its wealth of genotypic and phenotypic data collected in very large numbers, the UK Biobank study provides an excellent opportunity to explore the causal role of SUA level across a broad spectrum of disease outcomes. In this study, we performed an MR-PheWAS in UK Biobank database to discover disease outcomes related to genetic variations of SUA level and to investigate if any association is causal.

## Methods

### UK Biobank data

The UK Biobank is a large-scale, population-based, prospective cohort that enrolled over 500 000 participants aged 40–69 years. The recruited participants provided a wide range of self-reported baseline information. Blood samples were collected for biochemical tests and genotyping. Their national health records have been linked with the baseline and genotypic data for longitudinal follow-up. Genotypic and phenotypic data used in this study were obtained from UK Biobank under an approved data request application (application ID: 10775).

### Genotyping and quality control

Genotyping, quality control and genetic imputation were performed by the UK Biobank team prior to the interim release of genotypic data for 150 000 participants. The procedure of genotyping and quality control is presented in detail at https://biobank.ctsu.ox.ac.uk/crystal/docs/genotyping_qc.pdf. We used the field variables made available by the UK Biobank for quality control to exclude the samples that had high missingness or heterozygosity, outlying short runs of homozygosity, and sex mismatch (see online supplementary table S1). We constrained our analyses to participants who were self-reported British and confirmed to be Caucasians based on the genetic principal component analysis performed by the UK Biobank. The quality control process generated a genotypic data set output with 120 091 individuals included in the current analysis.

10.1136/annrheumdis-2017-212534.supp1 Supplementary data

### Phenotyping and mapping ICD-10 or ICD-9 to phecode

We focused on phenotypes in relation to diagnostic disease outcomes. We analysed two phenotypic data sets (inpatient hospital episode records and cancer registry data) in the UK Biobank using the phecode schema (see online supplementary text for phenotyping and mapping process).^21^ The coding for clinical diagnoses in these data sets followed the WHO’s International Classification of Diseases (ICD) coding systems, but used different ICD versions (ICD-10 or ICD-9) according to the date of record. We included both ICD-10 and ICD-9 codes to define the case and control groups. Since cancer registry data overlapped with the cancer diagnosis in inpatient hospital records, we pooled the cancer registry data into the hospital episode data as a complement to the cancer diagnosis.

10.1136/annrheumdis-2017-212534.supp2 Supplementary data

### Statistical analysis

The statistical analysis included three main steps: first, we performed a PheWAS to identify disease outcomes that were associated with genetic risk loci of SUA level; second, we performed MR analysis by using both the inverse-variance weighted (IVW) method and MR Egger approach to explore causal relationship for identified PheWAS asscoations^22 23^; and third, we applied HEIDI (heterogeneity in dependent instruments) test to exclude the cross-phenotype associations caused by genetic linkage.^24^

#### Genetic instruments

We selected 31 SUA-associated single nucleotide polymorphisms (SNPs) as genetic instruments (see online supplementary table S2), which were previously reported to be independently associated with SUA level in genome-wide association studies (GWAS).^25 26^ We obtained the SNP effect on SUA level from the largest GWAS performed in European population.^25^ The overall proportion of variance (R^2^ of SUA level explained by the selected genetic instruments) was estimated to be close to 7.0%.^25^

#### Phenome-wide association analysis

In phenome-wide analysis, we used 31 SUA-associated SNPs as genetic instruments individually to scan across a wide range of disease outcomes defined by the phecode system.^21^ With the PheWAS algorithm,^27^ a series of case–control tests was performed: (1) the case group was generated by including patients with the tested phecode; (2) participants were assigned to the control group based on the absence of both the tested phecode and related phecodes (patients who had the parent, child or sibling phecodes of the tested phecode were excluded from the control group)^27^; and (3) to ensure statistical power, analysis was only performed for phecode with no less than 200 cases. This minimum number of cases was suggested based on a simulation of power estimates for PheWAS analysis.^28^ We used logistic regression to test the associations between 31 individual genetic instruments (assuming an additive genetic model) and each phecode (number of cases ≥200) after adjusting for multiple covariates, including sex, body mass index (BMI), age, assessment centre and the principal components. Considering many phecodes were not independent, we used the false discovery rate (FDR) method to account for multiple testing.^29^

#### MR IVW, MR Egger and HEIDI test

We then explored the identified PheWAS associations in three possible scenarios (see online supplementary figure S1): (1) causality: the observed association was causal (through the SUA pathway); (2) pleiotropy: the observed association was due to pleiotropic effect of one causal variant (ie, linked to SUA level and the particular disease outcome through pleiotropy); and (3) genetic linkage: the observed association was caused by the linkage disequilibrium (LD) between two distinct causal variants, with one affecting SUA level and the other affecting the disease outcome.

10.1136/annrheumdis-2017-212534.supp3 Supplementary data

##### MR IVW

To explore if there was any causal effect on identified disease outcomes, we performed the conventional MR analysis by pooling the individual effect of each SNP using the IVW method to estimate the overall causal effect (see online supplementary text).^30^

##### MR Egger

We then performed MR Egger to attempt to correct for any potential pleiotropic effect in the causal estimates. This approach is applied to balance the pleiotropic effects derived from multiple genetic instruments (see online supplementary text).^23^

##### HEIDI test

We calculated HEIDI statistics for the SUA genetic loci that were associated with more than one disease outcome. This test was to examine if the cross-phenotype association was due to genetic linkage (see online supplementary text).^24^

### Sex stratification analysis

To account for any sex difference, we performed PheWAS and MR analyses in men and women separately. The sex-specific effects of SNPs on SUA level (see online supplementary table S2) were taken from the summary-level GWAS data provided by Köttgen *et al*.^25^

## Results

A total of 120 091 UK Biobank participants were included in the analysis, consisting of 56 845 men and 63 246 women with a mean age of 64.86 years in 2016 (SD of 7.95) (see online supplementary table S3). Within phenotypic data sets, we identified 684 324 hospital episodes and 23 174 cancer registration records, which included 7990 unique ICD-10 codes and 1998 unique ICD-9 codes. After mapping diagnostic ICD-10 or ICD-9 codes to phecodes, the phenotypic data consisted of 1807 distinct phecodes. After filtering out disease outcomes with low prevalence (number of cases <200), 568 phecodes (median number of cases=694 (range: 200–39 142)) were included in PheWAS analysis. These 568 phecodes were classified into 17 broadly related disease categories (table 1). We noted that the distribution of phenotypes examined was skewed across the different disease categories (see online supplementary figure S2), in which a large number of disease phenotypes were included in digestive, circulatory, endocrine and metabolic systems, but some disease categories, for example congenital anomalies, were not well represented in the study population.

10.1136/annrheumdis-2017-212534.supp4 Supplementary data

**Table 1:** The number of phenotypes and the number of cases in each disease category

Disease categories	Phenotypes (n)	Cases (n)			
		Minimum	Median	Mean	Maximum
Circulatory system	61	221	665	2937	39 142
Congenital anomalies	6	206	265	302	522
Dermatological diseases	24	201	706	2736	32 738
Diseases in sense organs	34	201	425	1216	11 306
Digestive diseases	73	201	949	2176	23 129
Neoplasms	59	203	763	1916	30 101
Infectious diseases	16	205	787	975	3192
Endocrine and metabolic diseases	25	229	492	2304	13 592
Haematopoietic diseases	10	205	1187	1600	3669
Neurological diseases	21	229	452	1282	11 828
Respiratory diseases	38	219	712	1713	19 238
Mental disorders	18	205	673	1926	8942
Genitourinary diseases	77	200	666	1606	29 859
Pregnancy complications	11	227	360	707	2531
Musculoskeletal diseases	44	263	1076	2482	21 822
Clinical symptoms	14	267	1237	2570	12 287
Injuries and poisonings	37	211	589	911	4842

### Phenome-wide association analysis

The PheWAS analysis performed 17 608 case–control tests, leading to an adjusted significance threshold of P<8.57e-05 corresponding to an FDR of q<0.05 to account for the multiple testing. A total of 27 pairs of genotype–phenotype associations passed the significance threshold of FDR correction (P<8.57e-05) in the overall PheWAS analysis with adjustment for covariates (table 2). Results of PheWAS without adjustment for BMI are shown in online supplementary table S4. The sex-stratified PheWAS analysis identified 10 pairs of genotype–phenotype association in men and 10 pairs of genotype–phenotype association in women (see online supplementary table S5). When compared with the overall PheWAS analysis, five new pairs of association were identified from the sex-stratified PheWAS analysis (see online supplementary table S5).

**Table 2:** Genotype–phenotype associations identified from PheWAS after correcting multiple testing by FDR (P<8.57e-05)

Phecode	Description	SNP_risk allele*	Allele frequency	Total (n)	Cases (n)	OR (95% CI)	P†
274.1	Gout	rs2231142_T	0.11	119 555	1003	1.89 (1.69 to 2.12)	5.41e-28
275.1	Disorders of iron metabolism	rs1165151_G	0.45	119 063	205	3.56 (2.78 to 4.56)	1.41e-23
244.4	Hypothyroidism	rs653178_C	0.48	118 821	4146	1.21 (1.16 to 1.27)	3.90e-17
246	Disorders of thyroid	rs653178_C	0.48	119 601	4926	1.18 (1.14 to 1.23)	8.82e-16
274.1	Gout	rs12498742_A	0.23	118 960	1002	1.54 (1.37 to 1.74)	7.94e-13
275.1	Disorders of iron metabolism	rs742132_A	0.29	119 271	205	2.80 (2.10 to 3.74)	3.13e-12
401	Hypertensive disease	rs653178_C	0.48	119 762	23 634	1.06 (1.04 to 1.09)	1.68e-08
401.1	Essential hypertension	rs653178_C	0.48	119 688	23 560	1.06 (1.04 to 1.09)	2.00e-08
411.4	Coronary atherosclerosis	rs653178_C	0.48	119 460	9526	1.09 (1.05 to 1.12)	1.27e-07
411	Ischaemic heart disease	rs653178_C	0.48	119 401	9467	1.09 (1.05 to 1.12)	1.33e-07
211	Benign neoplasm of digestive system	rs11264341_C	0.43	117 030	1504	0.83 (0.77 to 0.89)	2.41e-07
274.1	Gout	rs1260326_T	0.39	119 555	1003	1.26 (1.15 to 1.38)	3.86e-07
459.9	Circulatory disease	rs653178_C	0.48	119 677	39 142	1.05 (1.03 to 1.06)	2.24e-06
411.2	Myocardial infarction	rs653178_C	0.48	113 559	3625	1.12 (1.07 to 1.18)	2.80e-06
557.1	Coeliac disease	rs1165151_G	0.45	99 783	549	1.33 (1.18 to 1.51)	4.30e-06
557.1	Coeliac disease	rs653178_C	0.48	99 965	550	1.31 (1.16 to 1.48)	9.28e-06
427.2	Atrial fibrillation and flutter	rs6598541_A	0.35	113 261	4333	1.11 (1.06 to 1.16)	9.92e-06
960	Poisoning by antibiotics	rs1165151_G	0.45	112 343	1027	0.82 (0.75 to 0.90)	1.22e-05
535	Gastritis and duodenitis	rs478607_G	0.15	115 386	5233	1.12 (1.07 to 1.19)	1.34e-05
411.3	Angina pectoris	rs653178_C	0.48	114 967	5033	1.09 (1.05 to 1.14)	3.01e-05
669	Complications of labour and delivery	rs729761_G	0.28	113 240	2376	1.17 (1.09 to 1.26)	3.78e-05
272.11	Hypercholesterolaemia	rs1260326_T	0.39	118 921	10 201	1.07 (1.03 to 1.10)	3.82e-05
366	Cataract	rs6770152_G	0.43	116 218	4567	1.09 (1.05 to 1.14)	4.14e-05
471	Nasal polyps	rs10821905_A	0.17	112 745	983	1.26 (1.13 to 1.40)	4.61e-05
454.1	Varicose veins of lower extremity	rs2231142_T	0.11	111 390	3204	0.84 (0.78 to 0.92)	5.79e-05
401	Hypertensive disease	rs2079742_T	0.13	115 659	22 832	1.07 (1.03 to 1.10)	7.00e-05
401.1	Essential hypertension	rs2079742_T	0.13	115 588	22 761	1.07 (1.03 to 1.10)	7.02e-05

*Effect allele was harmonised to be the SUA-raising allele defined by Köttgen *et al*.^25^

†Significance threshold of P<8.57e-05 corresponds to an FDR of q<0.05 after correcting the multiple testing.

FDR, false discovery rate; PheWAS, phenome-wide association study; SUA, serum uric acid.

These identified genotype–phenotype associations were distributed across 15 SUA genetic loci, of which 5 loci were associated with more than one disease outcome: rs653178 in *ATXN2/SH2B3* locus (number of disease outcomes: $n_{outcomes}=10$), rs1165151 in *SLC17A3* locus ($n_{outcomes}=3$), rs1260326 in *GCKR* locus ($n_{outcomes}=3$), rs2231142 in *ABCG2* locus ($n_{outcomes}=4$) and rs2079742 in *BCAS3* locus ($n_{outcomes}=2$). Of note, six disease outcomes shared genetic associations with SUA level at more than one locus: gout (number of loci: $n_{loci}=3$), inflammatory polyarthropathies ($n_{loci}=2$), disorders of iron metabolism ($n_{loci}=2$), coeliac disease ($n_{loci}=2$), hypertensive disease ($n_{loci}=2$) and essential hypertension ($n_{loci}=2$).

In summary, the PheWAS analyses identified 25 unique disease groups/outcomes (corresponding to 25 unique phecodes) that shared genetic risk loci with SUA level, which included 9 disease groups (inflammatory polyarthropathies, hypertensive disease, circulatory disease, disorders of metabolism, disorders of thyroid, other diseases of respiratory system, disorder of skin and subcutaneous tissue, benign neoplasm of digestive system, and complications of labour and delivery) and 16 specific disease outcomes (gout, essential hypertension, angina pectoris, myocardial infraction, coronary atherosclerosis, ischaemic heart disease, atrial fibrillation and flutter, varicose veins of lower extremity, hypercholesterolaemia, disorders of iron metabolism, coeliac disease, hypothyroidism, gastritis and duodenitis, poisoning by antibiotics, cataract, and nasal polyps). The mappings of ICD codes to these 25 phecodes and their hierarchical relationships are shown in online supplementary table S6.

### MR IVW, MR Egger and HEIDI test

We then performed MR analysis using the IVW method to explore if there was any causal link between SUA level and the 25 disease groups/outcomes identified from PheWAS analysis. The MR IVW analysis suggested a potential causal link for 7 out of 25 disease groups/outcomes. The corresponding effect estimate on each disease outcome is presented in table 3. It was indicated that genetically determined higher SUA level was potentially causally linked with an increased risk of three disease groups, including inflammatory polyarthropathies (OR=1.22, 95% CI 1.11 to 1.34, P=1.10e-04), hypertensive disease (OR=1.08, 95% CI 1.03 to 1.14, P=0.004) and disorders of metabolism (OR=1.07, 95% CI 1.01 to 1.14, P=0.03), and of four specific disease outcomes, including gout (OR=4.88, 95% CI 3.91 to 6.09, P=3.55e-15), essential hypertension (OR=1.08, 95% CI 1.03 to 1.14, P=0.005), myocardial infarction (OR=1.16, 95% CI 1.03 to 1.30, P=0.015) and coeliac disease (OR=1.41, 95% CI 1.05 to 1.89, P=0.02).

**Table 3:** PheWAS associations assessed by conventional MR IVW and MR Egger analysis

Disease outcomes	MR IVW			MR Egger			
	OR (95% CI)	P effect	Power*	OR (95% CI)	P effect	P pleiotropy	Power*
Gout	4.88 (3.91 to 6.09)	3.55e-15	1.00	4.58 (2.72 to 7.72)	1.76e-06	0.73	1.00
Inflammatory polyarthropathies†	1.22 (1.11 to 1.34)	1.10e-04	0.99	1.15 (1.01 to 1.31)	0.03	0.23	0.83
Essential hypertension	1.08 (1.03 to 1.14)	5.07e-03	0.82	0.93 (0.83 to 1.05)	0.23	1.13e-03	0.73
Hypertensive disease	1.08 (1.03 to 1.14)	4.23e-03	0.82	0.93 (0.83 to 1.05)	0.24	1.19e-03	0.73
Myocardial infarction	1.16 (1.03 to 1.30)	0.02	0.70	1.03 (0.84 to 1.27)	0.75	0.13	0.08
Coeliac disease	1.41 (1.05 to 1.89)	0.02	0.72	1.31 (0.68 to 2.54)	0.41	0.75	0.48
Disorders of metabolism†	1.07 (1.01 to 1.14)	0.03	0.52	1.01 (0.91 to 1.14)	0.80	0.18	0.06
Coronary atherosclerosis	1.07 (0.99 to 1.15)	0.08	0.41	0.99 (0.85 to 1.17)	0.95	0.20	0.06
Ischaemic heart disease	1.07 (0.99 to 1.15)	0.09	0.41	0.99 (0.85 to 1.16)	0.91	0.20	0.06
Angina pectoris	1.04 (0.94 to 1.15)	0.41	0.11	0.95 (0.80 to 1.12)	0.51	0.11	0.15
Atrial fibrillation and flutter	1.01 (0.91 to 1.12)	0.87	0.05	0.90 (0.75 to 1.08)	0.23	0.07	0.41
Circulatory disease	1.04 (1.00 to 1.09)	0.08	0.40	0.97 (0.89 to 1.07)	0.57	0.05	0.26
Varicose veins of lower extremity	0.86 (0.72 to 1.02)	0.09	0.55	0.86 (0.67 to 1.10)	0.24	0.97	0.55
Disorders of iron metabolism	1.19 (0.74 to 1.90)	0.45	0.11	0.79 (0.15 to 4.07)	0.77	0.47	0.12
Hypercholesterolaemia	1.14 (0.96 to 1.36)	0.12	0.94	1.18 (0.88 to 1.58)	0.27	0.78	0.99
Hypothyroidism	1.10 (0.99 to 1.23)	0.07	0.39	0.99 (0.75 to 1.32)	0.97	0.30	0.05
Disorders of thyroid	1.08 (0.98 to 1.20)	0.10	0.31	1.01 (0.79 to 1.29)	0.94	0.41	0.05
Benign neoplasm of digestive system	0.93 (0.78 to 1.10)	0.36	0.11	0.90 (0.64 to 1.26)	0.52	0.79	0.18
Gastritis and duodenitis	0.97 (0.88 to 1.07)	0.53	0.09	0.95 (0.80 to 1.13)	0.55	0.70	0.16
Nasal polyps	1.08 (0.88 to 1.34)	0.45	0.10	1.09 (0.73 to 1.60)	0.67	0.98	0.12
Cataract	0.99 (0.90 to 1.09)	0.85	0.05	0.91 (0.75 to 1.10)	0.34	0.23	0.36
Poisoning by antibiotics	0.85 (0.70 to 1.04)	0.14	0.25	1.00 (0.68 to 1.48)	1.00	0.28	0.05
Complications of labour and delivery†	0.89 (0.76 to 1.03)	0.12	0.30	0.78 (0.59 to 1.02)	0.08	0.20	0.83
Other diseases of respiratory system†	1.11 (0.94 to 1.31)	0.19	0.22	1.16 (0.92 to 1.46)	0.22	0.64	0.42
Disorder of skin and subcutaneous tissue†	0.99 (0.93 to 1.06)	0.77	0.06	0.98 (0.89 to 1.09)	0.75	0.85	0.09

*The statistical power of MR analyses was calculated by using the non-centrality parameter-based approach^65^; the overall proportion of variance (R^2^) of serum uric acid level explained by the genetic instruments was estimated to be 7.0%.^25^

†Disease outcomes identified from sex-stratified PheWAS analysis.

IVW, inverse-variance weighted; MR, Mendelian randomisation; PheWAS, phenome-wide association study.

To explore and correct for any possible pleiotropic effect of multiple instruments, we then conducted the MR Egger analysis (table 3). After balancing out the potential pleiotropic effects, the putative causal link of SUA level with gout (OR=4.58, 95% CI 2.72 to 7.72, $P_{effect}$= 1.76e-06) and its umbrella disease group, inflammatory polyarthropathies (OR=1.15, 95% CI 1.01 to 1.31, $P_{effect}$=0.03), remained statistically significant and there was no indication of unbalanced pleiotropy ($P_{pleiotropy}$=0.73 and $P_{pleiotropy}$=0.23, respectively). The putative causal effect of SUA level on the other five disease groups/outcomes was not statistically significant in the MR Egger model. The causal effects of each individual SNPs on these seven disease groups/outcomes are shown in online supplementary figures S3–S9. Unbalanced pleiotropy was observed for essential hypertension ($P_{pleiotropy}$=0.001) and its umbrella disease group, hypertensive disease ($P_{pleiotropy}$=0.001). For myocardial infarction, coeliac disease and disorders of metabolism, the putative causal effect was not statistically significant in the MR Egger model ($P_{effect}$=0.75, $P_{effect}$ =0.41 and $\;P_{effect}$=0.80, respectively), although there was no evidence of unbalanced pleiotropy ($P_{pleiotropy}$=0.13, $P_{pleiotropy}$ =0.75 and $P_{pleiotropy}$=0.18, respectively). The results of the sex-stratified MR IVW are presented in online supplementary table S7.

Finally, to distinguish the genotype–phenotype association of pleiotropy from LD, the HEIDI test was performed for the five genetic loci (rs653178 in *ATXN2/SH2B3* locus, rs1165151 in *SLC17A3* locus, rs1260326 in *GCKR* locus, rs2231142 in *ABCG2* locus and rs2079742 in *BCAS3* locus) that were associated with multiple disease outcomes in the PheWAS analysis (see online supplementary figures S10–S14). Based on the HEIDI test, we identified 14 disease outcomes that were associated with the SUA genetic risk loci due to pleiotropy (with $P_{HEIDI\;}$ >0.05). The strongest pleiotropic locus was the *ATXN2/SH2B3*, where three SNPs (rs653178, rs4766578 and rs3184504) in near-complete LD (r^2^=0.99) were tagged as the lead SNPs associated with 10 disease groups/outcomes as a cluster of cardiovascular diseases and autoimmune disorders (see online supplementary figure S10). Other potential pleiotropic effects included the associations of *BCAS3* locus (rs2079742) with essential hypertension ($P_{HEIDI\;}$=0.10) and hypertensive disease ($P_{HEIDI\;}$=0.09) (see online supplementary figure S11), the associations of *ABCG2* locus (rs2231142) with varicose veins of lower extremity ($P_{HEIDI\;}$=0.32) (see online supplementary figure S12), and the association of *SLC17A3* locus (rs1165151) with poisoning by antibiotics ($P_{HEIDI\;}$=0.26) (see online supplementary figure S13).

10.1136/annrheumdis-2017-212534.supp12 Supplementary data

10.1136/annrheumdis-2017-212534.supp13 Supplementary data

10.1136/annrheumdis-2017-212534.supp14 Supplementary data

10.1136/annrheumdis-2017-212534.supp15 Supplementary data

Our analysis rejected the null hypothesis of a pleiotropic model for the shared genetic association between SUA level and disorders of iron metabolism at the *SLC17A3* locus (rs1165151) ($P_{HEIDI\;}$=5.54e-28); we identified a different causal variant (rs17342717 in *SLC17A1*) that was in LD with the SNP rs1165151 (r^2^=0.24) and strongly associated with the disorders of iron metabolism (P=1.69e-129) (see online supplementary figure S13). Similarly, for the associations between the *SLC17A3* locus (rs1165151) and coeliac disease ($P_{HEIDI\;}$=6.51e-16) (see online supplementary figure S13), and the *GCKR* locus (rs1260326) and hypercholesterolaemia ($P_{HEIDI\;}$=3.27e-11) (see online supplementary figure S14), the pattern of shared regional genetic association was more consistent with a genetic linkage model, and the SNP with the smallest P value was tagged as an index of the distinct causal variant affecting the examined disease outcome.

10.1136/annrheumdis-2017-212534.supp16 Supplementary data

## Discussion

In PheWAS analysis by using SUA-associated SNPs as genetic instruments, we replicated the findings of the largest GWAS performed by Köttgen and the findings of the most recent candidate gene-based association study conducted in UK Biobank, which indicated that two SUA-related SNPs (rs12498742 in *SLC2A9* locus and rs2231142 in *ABCG2* locus) are significantly associated with gout at GWAS P value threshold (P<5.0e-08).^25 31^ We conducted a conventional MR analysis (using the IVW method) and an MR Egger analysis, which accounts for potential pleiotropic effects, to investigate potential causal links with SUA level. These both confirmed potential causal effects of SUA level on gout and inflammatory polyarthropathies. The latter category represents the disease group term that includes gout, and thus this finding may just reflect the causal role of SUA in gout. However, this study cannot exclude a causal association between SUA and other inflammatory polyarthropathies, and this may be worth further study. Given that many comorbidities are commonly reported in patients with gout, it is of interest to consider the evidence for SUA sharing genetic risk loci with some of these diseases, such as cardiovascular/metabolic diseases and autoimmune disorders, and the evidence for a possible causal role for SUA in these conditions.

Overall, we identified 32 pairs of genotype–phenotype associations, which covered a wide range of phenotypic categories including endocrine/metabolic diseases, cardiovascular diseases and autoimmune disorders. Our PheWAS analysis replicated 14 pairs of previously known genotype–phenotype (or closely related phenotypic groups) associations reported in the GWAS Catalog (see online supplementary table S2 and table 2). For example, rs653178 (*ATXN2/SH2B3* locus) was previously reported to be associated with diastolic blood pressure,^32^ myocardial infarction,^33^ peripheral artery disease,^34^ coeliac disease^35^ and serum thyroid peroxidase antibody levels.^36^ In our PheWAS, this SNP was statistically significantly associated with the same phenotypes (ie, coeliac disease, myocardial infarction) or similar phenotypic groups (ie, hypertension, circulatory and heart diseases, hypothyroidism and other disorders of thyroid). We also identified 18 novel genotype–phenotype associations (at the PheWAS threshold of P<8.57e-05), of which the association between rs1165151 (*SLC17A3* locus) and disorders of iron metabolism had the smallest P value (P=1.23e-19).

We performed conventional MR analysis, using the IVW method, to investigate whether there was a potential causal link between SUA level and the 25 unique disease groups/outcomes identified from PheWAS. The results of MR IVW analysis suggested a potential causal effect of SUA level on three disease groups, including inflammatory polyarthropathies (as noted above), hypertensive disease and disorders of metabolism, and four specific individual disease outcomes, including gout (as noted above), essential hypertension, myocardial infarction and coeliac disease. When adopting the advanced MR Egger analysis to account for potential pleiotropic effects, it is indicated that, except for gout and inflammatory polyarthropathies, all the other putative causal associations suggested by MR IVW analysis were probably inflated by the presence of pleiotropy. However, although the MR Egger analysis is more robust in dealing with pleiotropy, this method is not infallible.^37^ Intuitively, the genetic instrument with larger effect on SUA level is expected to have a larger effect on disease outcome and would exert stronger influence in the MR Egger regression model. With indepth examination of the individual SNP effects on SUA level against the SNP effects on disease outcomes (see online supplementary figures S5–S8), we found that the outlying variant (rs12498742 in *SLC2A9*) that had the strongest association with SUA level showed a negative (null) effect on essential hypertension and hypertensive disease, which reversed the sign of the overall putative causal effect and led to a rejection of the intercept test. Given the influence of the outlying variant, the unbalanced pleiotropy and relatively moderate statistical power (power=0.73), we would interpret that unbalanced pleiotropy between SUA level and hypertension is an issue for their causal inference in MR Egger analysis.

Previous observational studies have reported sex difference in the association between SUA level and the development of cardiovascular diseases,^38–42^ but few studies have addressed the sex difference by using MR approach to keep out the influence of environmental confounders. Our study identified a few more cardiovascular diseases (eg, coronary atherosclerosis, ischaemic heart disease) that were potentially causally linked with the genetic variation of SUA level in women, but not in men. These MR findings were concordant with results from observational studies, which indicated that the relationship between SUA level and cardiovascular disease was particularly strong in women, especially for heart disease.^41 43 44^ Although these putative causal associations specific to women were not verified by MR Egger, this may be due to the decreased statistical power of MR Egger (and a higher risk of type 2 error). The biological mechanism that can lead the association of SUA level with cardiovascular disease to be more pronounced in women than in men remains a matter for further investigation.

We also found that several PheWAS associations were likely driven by LD. For instance, the outstanding PheWAS association between disorders of iron metabolism and the SNP rs1165151 in *SLC17A3* locus was not consistent with a pleiotropic model, and further examination found the SUA-associated SNP rs1165151 was located in LD (r^2^=0.24), with the rs17342717 variant in *SLC17A1* locus, which was strongly associated with disorders of iron metabolism (P=1.69e-129). This SNP (rs17342717) is also associated with red blood cell traits and serum iron levels in previous GWAS.^45 46^ We suggest that the implications of these findings have wider relevance for PheWAS studies. Typically, associations of a single SNP with multiple phenotypes were claimed to be due to pleiotropy in previous PheWAS.^47 48^ However, as PheWAS focused on single variant without considering the correlations between SNPs, we would suggest that an additional examination of LD is necessary when we identify pleiotropic links.

In contrast, the pattern of shared regional genetic associations of SUA level with multiple disease outcomes at *ATXN2/S2HB3* locus was more consistent with a pleiotropic model, where we interpreted this locus influenced a cluster of cardiovascular diseases and autoimmune disorders. However within the *ATXN2/S2HB3* locus, there are three leading SNPs (rs653178, rs4766578 and rs3184504) in high LD (r^2^=0.99). In this case, we were unable to provide an indication of whether the observed associations are due to pleiotropy or genetic linkage, as it was difficult to infer the causal variant. Although SNP rs653178 was reported as the lead variant influencing SUA level at this locus in GWAS, the potential biological mechanism underlying this effect is unclear.^25^ Furthermore, although the implication of the rs653178 on the regulation of blood pressure, cardiovascular diseases and coeliac disease has been suggested by a few GWAS,^32–35^ a clear biological explanation for this role could not be demonstrated. Evidence from the functional follow-up of the *S2HB3* gene indicated that rs3184504 may be the causal variant, as the *S2HB3* gene encodes one of the S2HB family proteins, which have a diverse physiological roles on haematopoiesis, immune response and signalling, and variation in rs3184504 may introduce a new phosphorylation site affecting the function of the S2HB protein.^49 50^ We believe that further uncovering of the biological functions of this pleiotropic locus (eg, gene function follow-up, expression quantitative trait loci analysis) might be helpful to understand the complex underlying relationship of SUA level with cardiovascular and autoimmune diseases.

The sex-stratified MR IVW analysis identified that unspecified diseases in respiratory system were potentially causally linked with SUA level in women (with the MR Egger analysis showing a consistent causal effect). This finding is consistent with recently published experimental studies, which demonstrated that human airway epithelial cells and lung tissue expressed a functional UA production/secretion system and UA was crucial in mediating the development of allergic airway diseases and regulating the antigen-specific T cell proliferation.^51–54^ It was also speculated that fine, inhaled particulate matter can induce increased UA production in the human airway, which may contribute to allergic sensitisation and asthma pathogenesis.^55^ Evidence from other epidemiological studies suggested that high SUA level was associated with low lung function and high risk of respiratory symptoms and chronic obstructive pulmonary disease, but the direct causal relationship has not been established.^56–58^ Further investigation may be worth to explore the clinical relevance of SUA level in lung health and respiratory diseases.

Key strengths of our study included its potential to make novel discoveries in genotype–phenotype associations and to identify novel cross-phenotype associations, possibly reflecting common aetiology or causal mechanisms. Unlike the genome, for which genetic structure can be measured by reliable biological techniques, the definition of phenome varies across studies. Current published PheWAS have been limited primarily to billing ICD-9-clinical modification (CM) to phecode system, and the method for aggregating ICD-9-CM codes into phecodes has proven to be valuable in previous PheWAS studies.^21 59^ Our work broadened the utility of phecode system and illustrated the process of adopting phecode system in the updated ICD-10 version to define the phenome framework. Our mapping process revealed some potential shortcomings of the current phecode system (eg, the ICD-10 codes involving the personal or family history were missing elements in the phecode system), which should be improved as a future undertaking. Recent methodological applications (eg, tree-structured phenotypic model (TreeWAS)) can be applied in future PheWAS analyses.^60^ As we were preparing the manuscript for submission, a web resource within UK Biobank, the GeneATLAS, was released in the bioRxiv (prior to peer review).^61^ We checked our PheWAS findings in this database, but only 10 of the 31 SUA-related SNPs were included in their database (and associations with some disease outcomes were replicated for these SNPs).^61^ We focused on the causal relationships between SUA level and binary disease outcomes in MR analyses, and these findings were complementary to MR estimates of urate archived in the MR-Base database (http://eve.mrbase.org/), which mainly focused on quantitative traits.

On the other hand, our analysis was limited to phenotypes with no less than 200 cases; therefore, diseases with relatively low prevalence were not analysed. As the UK Biobank grows, we expect to perform PheWAS and MR analyses for more phenotypes, with the priority given to the ones of which the relationships with SUA level are much controversial, such as dementia.^62 63^ Furthermore for some analysed phenotypes, our PheWAS analysis may still have low power to detect small effect size. The use of the interim release of UK Biobank data and focusing on a very homogeneous population (self-reported British confirmed by principal component analysis (PCA)) limited the power of this study. Additionally, we did not analyse the self-reported UK Biobank data to avoid information bias, but this may have impacted on the comprehensiveness of PheWAS and have reduced the precision of MR estimates. To improve this limitation, we performed a sensitivity analysis for gout by comparing the MR estimates for hospital-diagnosed gout, self-reported gout and hospital-diagnosed/self-reported gout (see online supplementary table S8). The MR estimates were consistently statistically significant in any of the cases but with differences in their effect sizes. These differences might be due to the fact that gout cases ascertained from hospital discharge coding may be unrepresentative of gout, given hospitalised gout is more likely to be complicated by comorbidities, as reported by Robinson *et al*.^64^ While making efforts to dissect the PheWAS associations with different models, given the complexity of human genetic structure, these models are not mutually exclusive and each model has its own methodological limitations, thus strong conclusions are not always possible. Therefore, the realistic goal for the present study was to assess different lines of evidence (ie, causality, pleiotropy or genetic linkage) in order to characterise the identified PheWAS associations in relation to SUA level. It would be beneficial to assess whether measured SUA level, rather than its genetic proxy, is also associated with the observed disease outcomes, but data on the SUA biomarker are not yet available in UK Biobank.

Overall, this PheWAS analysis demonstrated that SUA level shares genetic risk loci with multiple disease outcomes, particularly cardiovascular/metabolic diseases and autoimmune disorders. These findings provide rationale for further investigation of whether these associations are causal. Our study indicated a putative causal effect of SUA level on three disease groups (inflammatory polyarthropathies, hypertensive disease and disorders of metabolism) and four specific disease outcomes (gout, essential hypertension, coeliac disease and myocardial infarction); when balancing out the pleiotropy, a robust conclusion about causality was made for gout and its encompassing disease group, inflammatory polyarthropathies. Unbalanced pleiotropy was identified as an issue for the causal inference on the association between SUA level and hypertension. Other potential causal relevance of SUA level with respiratory diseases is also worthy of further investigation. When interpreting the PheWAS associations from a view of pleiotropy, our analysis highlighted a key pleiotropic locus that influenced SUA level and multiple cardiovascular and autoimmune diseases. A further functional annotation of this locus might be helpful to understand the biological pathways that contribute to the phenotypic associations between SUA level and cardiovascular diseases (including hypertension).

10.1136/annrheumdis-2017-212534.supp5 Supplementary data

10.1136/annrheumdis-2017-212534.supp6 Supplementary data

10.1136/annrheumdis-2017-212534.supp7 Supplementary data

10.1136/annrheumdis-2017-212534.supp8 Supplementary data

10.1136/annrheumdis-2017-212534.supp9 Supplementary data

10.1136/annrheumdis-2017-212534.supp10 Supplementary data

10.1136/annrheumdis-2017-212534.supp11 Supplementary data
