# Supplementary material for: MR-PheWAS: exploring the causal effect of SUA level on multiple disease outcomes by using genetic instruments in UK Biobank
Source: Ann Rheum Dis. 2018 Feb 6;77(7):1039–47. doi: 10.1136/annrheumdis-2017-212534 (PMC6029646; doi:10.1136/annrheumdis-2017-212534)
Supplement: Supplementary data [file annrheumdis-2017-212534supp001.pdf]

Online Supplementary tables

Contents of supplementary tables:

Table S1: Sample quality control (QC) of genotype data in UK Biobank.

Table S2: Summary of the 31 SUA-associated SNPs identified from GWAS.

Table S3: Demographic characteristics of the sampled UK Biobank participants (n=120,091).

Table S4: Genotype-phenotype associations identified from PheWAS analysis without adjustment for BMI.

Table S5: Genotype-phenotype associations identified from sex-stratified PheWAS after correcting multiple testing by FDR.

Table S6: The mappings of ICD codes to phecodes for the 25 disease outcomes identified from PheWAS.

Table S7: Sex-stratified MR IVW analysis and MR Egger analysis.

Table S8: A sensitivity analysis of MR for gout cases defined by multiple criteria.

Table S1. Sample quality control (QC) of genotype data in UK Biobank.

Sample QC variables			Sample selection
**f.22050**	Pass or not	• This variable indicates the genotype quality for ~50,000 samples genotyped by UKBiLEVE array<br>• Sample quality control undertaken by Affymetrix was a DNA quality filter (dish quality control < 0.82) and initial clustering call rate (<97%);<br>• 520 duplicates passed Affymetrix quality control in both samples, 33 passed Affymetrix quality control in 1 sample, and 0 duplicates failed Affymetrix quality control in both samples.	Pass
**f.22051**	Pass or not	• This variable indicates QC steps performed for ~50,000 samples genotyped by UKBiLEVE array<br>• Sex mismatch (remove sample if submitted gender is different to gender inferred from sex chromosomes);<br>• Final call rate (<95%),<br>• Heterozygosity outliers (outlier if > 3sd from mean heterozygosity);<br>• Unintended duplicate (> 98% of alleles shared identical by descent);<br>• Ancestry principal components outlier (outlier if >10sd from mean on first 10 principal components).	Pass
**f.22010**	Pass or not	• This variable indicates the genotype quality for all samples;<br>• Samples showed signs of insufficient data quality;<br>• Individuals with high missingness or for which heterozygosity rates were not explained by the long runs of homozygosity (ROH) nor mixed ethnicity.	Pass
**Population structure**			
**f.22006**	Genetic ethnic group	• Ethnic groups determined by principle genetic components.	Select self-reported British and confirmed to be Caucasians by PCAs
**f.21000**	Ethnic background	• Self-reported ethnic background	
**f.22009**	Genetic principal components (PCAs)	• The first fifteen PCAs were available for the samples	
**Sex mismatch**			
**f.22001**	Genetic sex	• Sex inferred from X chromosome genotypes	Remove samples with sex mismatch
**f.31**	Self-reported sex	• Sex self-reported from the baseline assessment	

Table S2. Summary of the 31 SUA-associated SNPs identified from GWAS.

Genetic predictors				Overall effect			Men-specific effect			Women-specific effect			Other associated phenotypes reported in the human GWAS catalog
SNPs	Chr	Effect allele	Closest/CRAIL Gene	Beta	SE	p_val	Beta	SE	p_val	Beta	SE	p_val	
rs10480300	7	T	*PRKAG2/PRKAG2*	0.032	0.006	9.37E-07	0.043	0.010	1.70E-05	0.024	0.008	3.20E-03	Red blood cell traits[1]
rs10821905	10	A	*A1CF/ASAH2*	0.053	0.007	3.45E-12	0.042	0.011	3.80E-04	0.060	0.009	2.50E-10	None
rs11264341	1	C	*TRIM46/PKLR*	0.048	0.006	1.04E-14	0.055	0.009	1.10E-08	0.044	0.007	9.10E-09	None
rs1165151	6	G	*SLC17A1/SLC17A3*	0.092	0.005	4.52E-60	0.096	0.008	1.30E-28	0.089	0.007	4.20E-37	None
rs1171614	10	C	*SLC16A9/SLC16A9*	0.074	0.007	6.48E-23	0.086	0.011	1.90E-13	0.067	0.009	3.00E-13	Acylcarnitine levels,[2] Glycerophospholipid levels,[2] Blood metabolite levels[3]
rs1178977	7	A	*BAZ1B/MLXIPL*	0.050	0.007	6.68E-12	0.055	0.011	8.20E-07	0.046	0.009	2.60E-07	None
rs12498742	4	A	*SLC2A9/SLC2A9*	0.380	0.006	0.00E+00	0.269	0.010	6.40E-153	0.460	0.008	0.00E+00	Gout[4]
rs1260326	2	T	*GCKR/GCKR*	0.077	0.006	1.31E-40	0.091	0.009	3.00E-25	0.063	0.007	1.90E-04	Gout,[5] C-reactive protein levels,[6] Dyslipidemia,[7] Hematological and biochemical traits,[8] Chronic kidney disease,[9] Hypertriglyceridemia[10]
rs1394125	15	A	*UBE2Q2/NRG4*	0.043	0.006	9.78E-11	0.060	0.010	5.50E-06	0.032	0.008	1.00E-04	Chronic kidney disease[9], Kidney function[11]
rs1471633	1	A	*PDZK1/PDZK1*	0.061	0.005	1.40E-26	0.069	0.008	3.50E-15	0.054	0.007	1.60E-14	None
rs164009	17	A	*QRICH2/PRPSAP1*	0.029	0.006	7.06E-07	0.024	0.009	6.20E-03	0.032	0.007	8.20E-06	None
rs17050272	2	A	*INHBB/INHBB*	0.037	0.006	9.36E-09	0.049	0.010	6.50E-07	0.030	0.008	1.90E-04	Glomerular filtration rate(creatinine)[11]
rs17632159	5	G	*TMEM171/TMEM171*	0.038	0.006	2.00E-09	0.043	0.010	1.30E-05	0.039	0.008	1.10E-06	None
rs17786744	8	G	*STC1/STC1*	0.031	0.005	8.82E-08	0.033	0.009	2.10E-04	0.029	0.007	2.10E-04	None
rs2078267	11	C	*SLC22A11/SLC22A11*	0.078	0.006	8.73E-36	0.085	0.009	2.90E-19	0.071	0.007	5.70E-20	Gout,[12] Cardiovascular disease risk factors[12]
rs2079742	17	T	*BCAS3/C17orf82*	0.051	0.008	6.24E-09	0.054	0.013	5.60E-05	0.048	0.010	1.00E-05	Metabolite levels (small molecules and protein measures)[13]
rs2231142	4	T	*ABCG2/ABCG2*	0.220	0.009	4.43E-116	0.270	0.014	3.80E-75	0.181	0.011	1.30E-52	Gout[4]
rs2307394	2	C	*ORC4L/ACVR2A*	0.035	0.006	7.26E-09	0.036	0.009	1.20E-04	0.034	0.007	4.70E-06	None
rs2941484	8	T	*HNF4G/HNF4G*	0.049	0.006	3.91E-17	0.048	0.009	6.20E-08	0.046	0.007	1.30E-10	None
rs3741414	12	C	*INHBC/INHBE*	0.071	0.007	9.79E-22	0.091	0.011	7.00E-16	0.057	0.009	4.30E-10	None
rs478607	11	G	*NRXN2/SLC22A12*	0.048	0.007	5.31E-10	0.058	0.012	9.60E-07	0.043	0.009	8.80E-06	None
rs642803	11	C	*OVOL1/LTBP3*	0.043	0.005	4.51E-14	0.047	0.008	8.00E-08	0.042	0.007	2.10E-09	None

rs653178	12	C	*ATXN2/SH2B3*	0.036	0.005	2.45E-10	0.044	0.009	7.50E-07	0.032	0.007	5.50E-06	Celiac disease,[14] Diastolic Blood Pressure,[15] Chronic kidney disease,[9] Serum thyroid peroxidase antibody levels,[16] Rheumatoid arthritis,[17] Peripheral artery disease,[18] Myocardial infarction,[19] Inflammatory bowel disease[20]
rs6598541	15	A	*IGF1R/IGF1R*	0.044	0.006	5.20E-13	0.039	0.009	2.70E-05	0.050	0.007	1.60E-11	None
rs675209	6	T	*RREB1/RREB1*	0.063	0.006	1.38E-21	0.060	0.010	3.30E-09	0.064	0.008	2.00E-15	Gout,[12] Cardiovascular disease risk factors[12]
rs6770152	3	G	*SFMBT1/MUSTN1*	0.048	0.006	2.66E-16	0.052	0.009	6.70E-09	0.047	0.007	6.00E-11	None
rs7188445	16	G	*MAF/MAF*	0.032	0.006	1.15E-07	0.025	0.009	7.90E-03	0.040	0.007	6.40E-08	None
rs7193778	16	C	*NFAT5/NFAT5*	0.047	0.008	2.36E-08	0.048	0.012	2.10E-04	0.045	0.010	1.00E-05	None
rs7224610	17	C	*HLF/HLF*	0.038	0.006	4.74E-11	0.043	0.009	9.00E-07	0.034	0.007	3.00E-06	None
rs729761	6	G	*VEGFA/VEGFA*	0.046	0.006	3.05E-12	0.047	0.010	3.20E-06	0.047	0.008	3.20E-06	None
rs742132	6	A	*LRRC16A/LRRC16A*	0.035	0.006	1.90E-08	0.035	0.006	1.90E-08	0.035	0.006	1.90E-08	Cardiovascular disease risk factors,[12] Hematological and biochemical traits,[8] Metabolite levels[21]

Abbreviations: SNP=Single Nucleotide Polymorphism; GWAS= Genome Wide Association Study; GRAIL=Gene Recognition and Analysis Internet Link.

Table S3. Demographic characteristics of the sampled UK Biobank participants (n=120,091).

**Continuous variable**	**Mean (S.D.)**	**Number of missingness**
Age#	64.86 (7.95) years	0
Standing height	168.79 (9.20) cm	200
Weight	78.68 (16.08) kg	288
BMI†	27.54 (4.83) kg/m^2	329
**Categorical variable**	**Levels**	**Number of participants**
Sex	Male	56,845
	Female	63,246
Assessment centre	Barts	2,004
	Birmingham	5,427
	Bristol	10,808
	Bury	7,751
	Cardiff	4,539
	Croydon	4,933
	Edinburgh	4,257
	Glasgow	4,783
	Hounslow	5,082
	Leeds	11,188
	Liverpool	8,404
	Manchester	3,278
	Middlesborough	5,563
	Newcastle	9,592
	Nottingham	8,429
	Oxford	3,163
	Reading	7,021
	Sheffield	7,779
	Stockport(pilot)	88
	Stoke	5,229
	Swansea	571
	Wrexham	202

# Variable represents the mean age of participants in 2016, at the year when we assessed their medical records.
†BMI (body mass index) was calculated as the weight divided by the square of the height.

Table S4. Genotype-phenotype associations identified from PheWAS analysis without adjustment for BMI.

Phecode	Description	SNP_risk allele	n_total	n_cases	allele_freq	HWE_p	beta	SE	OR (95%CI)	P
274.1	Gout	rs2231142_T	119884	1008	0.113	0.642	0.619	0.057	1.86 (1.66, 2.08)	4.17E-27
275.1	Disorders of iron metabolism	rs1165151_G	119389	205	0.451	0.920	1.270	0.127	3.56 (2.78, 4.57)	1.34E-23
244.4	Hypothyroidism NOS	rs653178_C	119145	4169	0.482	0.690	0.184	0.023	1.20 (1.15, 1.26)	3.19E-16
246	Other disorders of thyroid	rs653178_C	119929	4953	0.483	0.708	0.163	0.021	1.18 (1.13, 1.23)	4.56E-15
274.1	Gout	rs12498742_A	119284	1007	0.233	0.773	0.429	0.060	1.54 (1.37, 1.73)	1.00E-12
275.1	Disorders of iron metabolism	rs742132_A	119598	205	0.293	0.790	1.029	0.148	2.80 (2.10, 3.74)	3.15E-12
211	Benign neoplasm of other parts of digestive system	rs11264341_C	117350	1507	0.429	0.949	-0.189	0.037	0.83 (0.77, 0.89)	2.68E-07
411.8	Other chronic ischemic heart disease	rs653178_C	119699	9495	0.483	0.720	0.075	0.016	1.08 (1.05, 1.11)	1.24E-06
411.4	Coronary atherosclerosis	rs653178_C	119787	9583	0.483	0.716	0.075	0.015	1.08 (1.05, 1.11)	1.41E-06
411	Ischemic Heart Disease	rs653178_C	119728	9524	0.483	0.723	0.075	0.015	1.08 (1.05, 1.11)	1.47E-06
274.1	Gout	rs1260326_T	119884	1008	0.393	0.813	0.218	0.045	1.24 (1.14, 1.36)	1.48E-06
401	Hypertension	rs653178_C	120091	23755	0.483	0.720	0.049	0.011	1.05 (1.03, 1.07)	3.59E-06
427.2	Atrial fibrillation and flutter	rs6598541_A	113564	4368	0.353	0.549	0.106	0.023	1.11 (1.06, 1.16)	3.69E-06
401.1	Essential hypertension	rs653178_C	120017	23681	0.483	0.707	0.049	0.011	1.05 (1.03, 1.07)	4.20E-06
557.1	Celiac disease	rs1165151_G	100035	550	0.450	0.904	0.285	0.062	1.33 (1.18, 1.50)	5.11E-06
960	Poisoning by antibiotics	rs1165151_G	112628	1031	0.451	0.864	-0.199	0.045	0.82 (0.75, 0.89)	8.01E-06
557.1	Celiac disease	rs653178_C	100218	551	0.482	0.306	0.271	0.061	1.31 (1.16, 1.48)	8.27E-06
411.2	Myocardial infarction	rs653178_C	113854	3650	0.482	0.603	0.105	0.024	1.11 (1.06, 1.16)	1.31E-05
535	Gastritis and duodenitis	rs478607_G	115704	5252	0.152	0.914	0.114	0.027	1.12 (1.06, 1.18)	2.50E-05
459.9	Circulatory disease NEC	rs653178_C	120005	39322	0.483	0.702	0.037	0.009	1.04 (1.02, 1.06)	4.54E-05
471	Nasal polyps	rs10821905_A	113054	986	0.172	0.725	0.227	0.056	1.26 (1.12, 1.40)	5.06E-05
366	Cataract	rs6770152_G	116535	4592	0.427	0.063	0.087	0.022	1.09 (1.05, 1.14)	6.44E-05
292.1	Aphasia/speech disturbance	rs164009_A	116338	425	0.385	0.969	0.291	0.074	1.34 (1.16, 1.55)	8.38E-05

Table S5. Genotype-phenotype associations identified from sex-stratified PheWAS after correcting multiple testing by FDR.*

Phecode	Description	SNP_effect _allele	n_total	n_cases	allele_freq	hwe_p	Beta	SE	OR (95%CI)	P
**Significant associations identified from PheWAS analysis in men**										
274.1	Gout	rs2231142_T	56528	885	0.113	0.939	0.681	0.061	1.98 (1.75, 2.23)	8.23E-29
274.1	Gout	rs12498742_A	56253	884	0.234	0.876	-0.430	0.064	1.54 (1.36, 1.74)	2.68E-11
714	Inflammatory polyarthropathies	rs2231142_T	56306	2685	0.113	0.956	0.209	0.042	1.23 (1.14, 1.34)	5.82E-07
274.1	Gout	rs1260326_T	56528	885	0.393	0.554	0.238	0.049	1.27 (1.15, 1.40)	1.01E-06
401	Hypertensive disease	rs653178_C	56668	13027	0.482	0.834	0.073	0.015	1.08 (1.04, 1.11)	1.34E-06
401.1	Essential hypertension	rs653178_C	56630	12989	0.482	0.824	0.073	0.015	1.08 (1.04, 1.11)	1.42E-06
277	Disorders of metabolism‡	rs3741414_C	56503	8017	0.243	0.604	-0.096	0.021	1.10 (1.06, 1.15)	3.98E-06
714	Inflammatory polyarthropathies‡	rs1260326_T	56306	2685	0.393	0.657	0.120	0.029	1.13 (1.07, 1.19)	3.09E-05
512	Other diseases of respiratory system‡	rs1471633_A	55070	977	0.462	0.930	-0.193	0.046	0.82 (0.75, 0.90)	3.25E-05
274.1	Gout	rs642803_C	55592	872	0.460	0.573	-0.198	0.050	1.22 (1.11, 1.34)	6.17E-05
**Significant associations identified from PheWAS analysis in women**										
244.4	Hypothyroidism NOS	rs653178_C	62363	3362	0.483	0.030	0.205	0.025	1.23 (1.17, 1.29)	7.95E-16
246	Other disorders of thyroid	rs653178_C	62970	3969	0.483	0.096	0.179	0.023	1.20 (1.14, 1.25)	2.51E-14
211	Benign neoplasm of digestive system	rs11264341_C	61908	896	0.431	0.872	0.257	0.048	0.77 (0.70, 0.85)	7.47E-08
411.4	Coronary atherosclerosis	rs653178_C	62971	2997	0.483	0.185	0.113	0.027	1.12 (1.06, 1.18)	2.71E-05
411	Ischemic Heart Disease	rs653178_C	62954	2980	0.483	0.157	0.112	0.027	1.12 (1.06, 1.18)	3.35E-05
669	Complications of labor and delivery#	rs729761_G	59622	2376	0.283	0.958	-0.159	0.039	1.17 (1.09, 1.27)	3.78E-05
401.1	Essential hypertension	rs2079742_T	60918	10237	0.136	0.339	-0.098	0.024	1.10 (1.05, 1.16)	5.35E-05
689	Disorder of skin and subcutaneous tissue#	rs2231142_T	63094	6142	0.113	0.349	-0.126	0.031	0.88 (0.83, 0.94)	5.72E-05
401	Hypertensive disease	rs2079742_T	60952	10271	0.136	0.310	-0.098	0.024	1.10 (1.05, 1.16)	5.78E-05
535	Gastritis and duodenitis	rs478607_G	60962	2845	0.153	0.790	0.144	0.036	1.15 (1.08, 1.24)	7.46E-05

*Significance threshold of $p<8.57e-05$ corresponds to a FDR of $q<0.05$ after correcting the multiple testing.

‡ Genotype-phenotype associations that were not identified in PheWAS analysis of overall population.

# Genotype-phenotype association that were not identified in PheWAS analysis of overall population.

Table S6. The mappings of ICD codes to phecodes for the 25 disease outcomes identified from PheWAS.

Phecode	ICD9/10	Descriptions
**274.1**	**Gout**	
ICD-9	274	Gout
	274	Gouty arthropathy
	274.1	Gouty nephropathy
	274.8	Gout with other specified manifestations
	274.9	Gout, unspecified
ICD-10	M10	Gout
	M10.0	Idiopathic gout
	M10.1	Lead-induced gout
	M10.2	Drug-induced gout
	M10.3	Gout due to impairment of renal function
	M10.4	Other secondary gout
	M10.9	Gout, unspecified
**714**	**Inflammatory polyarthropathies**	
ICD-9	714	Rheumatoid arthritis and other inflammatory polyarthropathies
	714	Rheumatoid arthritis
	714.1	Felty's syndrome
	714.2	Other rheumatoid arthritis with visceral or systemic involvement
	714.3	Juvenile chronic polyarthritis
	714.4	Chronic postrheumatic arthropathy
	714.8	Other specified inflammatory polyarthropathies
	714.9	Unspecified inflammatory polyarthropathy
ICD-10	M05	Seropositive rheumatoid arthritis
		M05.0-M05.9
	M06	Other rheumatoid arthritis
		M06.0-M06.9
	M07	Psoriatic and enteropathic arthropathies
		M07.0-M07.6
	M08	Juvenile arthritis
		M08.0-M08.9
	M09	Juvenile arthritis in diseases classified elsewhere
		M09.0-M09.8
	M10	Gout
		M10.0-M10.9
	M11	Other crystal arthropathies
		M11.0-M11.9
	M12	Other specific arthropathies
		M12.0-M12.8
	M13	Other arthritis
		M13.0-M13.9
	M14	Arthropathies in other diseases classified elsewhere
		M14.0-M14.8

**401.1**	**Essential hypertension**	
ICD-9	401	Essential hypertension
		401.0-401.9
ICD-10	I10	Essential (primary) hypertension
**401**	**Hypertensive disease**	
ICD-9	401	Essential hypertension
		401.0-401.9
	402	Hypertensive heart disease
		402.0-402.9
	403	Hypertensive chronic kidney disease
		403.0-403.9
	404	Hypertensive heart and chronic kidney disease
		404.0-404.9
	405	Secondary hypertension
		405.0-405.9
ICD-10	I10	Essential (primary) hypertension
	I11	Hypertensive heart disease
		I11.0-I11.9
	I12	Hypertensive renal disease
		I12.0-I12.9
	I13	Hypertensive heart and renal disease
		I13.0-I13.9
	I15	Secondary hypertension
		I15.0-115.9
**411.4**	**Coronary atherosclerosis**	
ICD-9	411.81	Acute coronary occlusion without myocardial infarction
	414	Other forms of chronic ischemic heart disease
		414.00-414.07
	414.1	Aneurysm and dissection of heart
		414.10-414.19
	414.2	Chronic total occlusion of coronary artery
	414.3	Coronary atherosclerosis due to lipid rich plaque
	996.03	Due to coronary bypass graft
	V45.81	Aortocoronary bypass status
	V45.82	Percutaneous transluminal coronary angioplasty status
ICD-10	I20	Angina pectoris
		I20.0-I20.9
	I21	Acute myocardial infarction
		I21.0-I21.9
	I22	Subsequent myocardial infarction
		I22.0-I22.9
	I23	Certain current complications following acute myocardial infarction
		I23.0-I23.9
	I24	Other acute ischaemic heart diseases

		I24.0-I24.9
	I25	Chronic ischaemic heart disease
		I25.0-I25.9
	Z95.1	Presence of aortocoronary bypass graft
	Z95.5	Presence of coronary angioplasty implant and graft
**411.2**	**Myocardial infarction**	
ICD-9	410	Acute myocardial infarction
		410.0-410.9
	411	Postmyocardial infarction syndrome
	412	Old myocardial infarction
	429.7	Certain sequelae of myocardial infarction, not elsewhere classified
		429.71-429.79
ICD-10	I21	Acute myocardial infarction
		I21.0-I21.9
	I22	Subsequent myocardial infarction
		I22.0-I22.9
	I23	Certain current complications following acute myocardial infarction
		I23.0-I23.9
	I24.1	Dressler syndrome
	I25.2	Old myocardial infarction
	I51.0	Cardiac septal defect
	I51.3	Intracardiac thrombosis, not elsewhere classified
**427.2**	**Atrial fibrillation and flutter**	
ICD-9	427.3	Atrial fibrillation and flutter
		427.31-427.32
ICD-10	I48	Atrial fibrillation and flutter
		I48.0-I48.9
**411.3**	**Angina pectoris**	
ICD-9	413	Angina pectoris
		413.0-413.9
ICD-10	I20	Angina pectoris
		I20.0-I20.9
**411**	**Ischemic heart disease**	
ICD-9	410	Acute myocardial infarction
		410.0-410.9
	411	Other acute and subacute forms of ischemic heart disease
		411.0-411.8
	412	Old myocardial infarction
	413	Angina pectoris
		413.0-413.9
	414	Other forms of chronic ischemic heart disease
		414.0-414.9
	429	Ill-defined descriptions and complications of heart disease
	996.03	Due to coronary bypass graft

ICD-10	I20	Angina pectoris
		I20.0-I20.9
	I21	Acute myocardial infarction
		I21.0-I21.9
	I22	Subsequent myocardial infarction
		I22.0-I22.9
	I23	Certain current complications following acute myocardial infarction
		I23.0-I23.9
	I24	Other acute ischaemic heart diseases
		I24.0-I24.9
	I25	Chronic ischaemic heart disease
		I25.0-I25.9
**454.1**	**Varicose veins of lower extremity**	
ICD-9	454	Varicose veins of lower extremities
		454.0-454.9
ICD-10	I83	Varicose veins of lower extremities
		I83.0-I83.9
**459.9**	**Circulatory disease**	
ICD-9	459	Other disorders of circulatory system
		459.0-459.9
ICD-10	I00-I02	Acute rheumatic fever
	I05-I09	Chronic rheumatic heart diseases
	I10-I15	Hypertensive diseases
	I20-I25	Ischaemic heart diseases
	I26-I28	Pulmonary heart disease and diseases of pulmonary circulation
	I30-I52	Other forms of heart disease
	I60-I69	Cerebrovascular diseases
	I70-I79	Diseases of arteries, arterioles and capillaries
	I80-I89	Diseases of veins, lymphatic vessels and lymph nodes, not elsewhere classified
**244.4**	**Hypothyroidism NOS**	
ICD-9	244.9	Unspecified hypothyroidism
ICD-10	E03.9	Hypothyroidism, unspecified
**246**	**Disorders of thyroid**	
ICD-9	246	Disorders of thyrocalcitonin secretion
		246.0-246.9
ICD-10	E00	Congenital iodine-deficiency syndrome
		E00.0-E00.9
	E01	Iodine-deficiency-related thyroid disorders and allied conditions
		E01.1-E01.8
	E02	Subclinical iodine-deficiency hypothyroidism
	E03	Other hypothyroidism
		E03.0-E03.9
	E04	Other nontoxic goitre
		E04.0-E04.9

	E05	Thyrotoxicosis [hyperthyroidism]
		E05.0-E05.9
	E06	Thyroiditis
		E06.0-E06.9
	E07	Other disorders of thyroid
		E07.0-E07.9
**557.1**	**Celiac disease**	
ICD-9	579	Celiac disease
	579.1	Tropical sprue
ICD-10	K90.0	Celiac disease
	K90.1	Tropical sprue
**275.1**	**Disorders of iron metabolism**	
ICD-9	275	Disorders of iron metabolism
ICD-10	E83.1	Disorders of iron metabolism
**272.11**	**Hypercholesterolemia**	
ICD-9	272	Pure hypercholesterolemia
ICD-10	E78.0	Pure hypercholesterolemia
**277**	**Disorders of metabolism**	
ICD-9	277	Other and unspecified disorders of metabolism
		277.0-277.9
	783.9	Other symptoms concerning nutrition, metabolism, and development
	794.7	Basal metabolism
ICD-10	C96.0	Multifocal and multisystemic (disseminated) Langerhans-cell histiocytosis
	E43	Unspecified severe protein-energy malnutrition
	E70	Disorders of aromatic amino-acid metabolism
		E70.1-E70.9
	E71	Disorders of branched-chain amino-acid metabolism and fatty-acid metabolism
	E72	Other disorders of amino-acid metabolism
	E73	Lactose intolerance
	E74	Other disorders of carbohydrate metabolism
	E75	Disorders of sphingolipid metabolism and other lipid storage disorders
	E76	Disorders of glycosaminoglycan metabolism
	E77	Disorders of glycoprotein metabolism
	E78	Disorders of lipoprotein metabolism and other lipidaemias
	E79	Disorders of purine and pyrimidine metabolism
	E80	Disorders of porphyrin and bilirubin metabolism
	E83	Disorders of mineral metabolism
	E84	Cystic fibrosis
	E85	Amyloidosis
	E86	Volume depletion
	E87	Other disorders of fluid, electrolyte and acid-base balance
	E88	Other metabolic disorders
	E89	Postprocedural endocrine and metabolic disorders, not elsewhere classified
	E90	Nutritional and metabolic disorders in diseases classified elsewhere

**366**	**Cataract**	
ICD-9	366	Cataract
		366.0-366.9
	998.82	Cataract fragments in eye following cataract surgery
ICD-10	V43.1	Lens
	V45.61	Cataract extraction status
	H26	Other cataract
		H26.0-H26.9
	H28	Cataract and other disorders of lens in diseases classified elsewhere
		H28.0-H28.8
	Z96.1	Presence of intraocular lens
**960**	**Poisoning by antibiotics**	
ICD-9	960	Poisoning by antibiotics
		960.1-960.9
	961.8	Other anti-mycobacterial drugs
	V14.0	Personal history of allergy to Penicillin
	V14.1	Personal history of allergy to other antibiotic agent
	E856	Accidental poisoning by antibiotics
	E930	Adverse effects in therapeutic use of antibiotics
		E930.0- E930.9
	E931.8	Adverse effects in therapeutic use of anti-mycobacterial drugs
	E933.4	Adverse effects in therapeutic use of Penicillinase
ICD-10	T36	Poisoning by systemic antibiotics
		T36.5- T36.9
	T37.1	Poisoning by anti-mycobacterial drugs
	T37.8	Poisoning by other specified systemic anti-infectives and antiparasitics
	Z88.1	Personal history of allergy to other antibiotic agents
**471**	**Nasal polyps**	
ICD-9	471	Nasal polyps
		471.0-471.9
ICD-10	J33	Nasal polyp
		J33.0-J33.9
**512**	**Other diseases of respiratory system**	
ICD-9	519	Other diseases of respiratory system
		519.0-519.9
	786	Symptoms involving respiratory system and other chest symptoms
		786.0-786.9
ICD-10	J95	Postprocedural respiratory disorders, not elsewhere classified
		J95.0-J95.9
	J96	Respiratory failure, not elsewhere classified
		J96.0-J96.9
	J98	Other respiratory disorders
		J98.0-J98.9
	J99	Respiratory disorders in diseases classified elsewhere

		J99.0-J99.9
**535**	**Gastritis and duodenitis**	
ICD-9	535	Gastritis and duodenitis
		535.0-535.7
ICD-10	K29	Gastritis and duodenitis
		K29.0-K29.9
**211**	**Benign neoplasm of digestive system**	
ICD-9	211	Benign neoplasm of other parts of digestive system
		211.0-211.9
ICD-10	D13	Benign neoplasm of other and ill-defined parts of digestive system
		D13.0-D13.9
	D19.1	Mesothelial tissue of peritoneum
	K31.7	Polyp of stomach and duodenum
**669**	**Complications of labor and delivery**	
ICD-9	667	Retained placenta without hemorrhage
	669	Other complications of labor and delivery, not elsewhere classified
		669.0-669.9
	674	Other and unspecified complications of the puerperium, not elsewhere classified
		674.0-674.9
	677	Late effect of complication of pregnancy, childbirth, and the puerperium
ICD-10	O26.5	Maternal hypotension syndrome
	O60	Preterm labour and delivery
		O60.0- O60.9
	O61	Failed induction of labour
		O61.0- O61.9
	O62	Abnormalities of forces of labour
		O62.0- O62.9
	O63	Long labour
		O63.0- O63.9
	O64	Obstructed labour due to malposition and malpresentation of fetus
		O64.0- O64.9
	O65	Obstructed labour due to maternal pelvic abnormality
		O65.0- O65.9
	O66	Other obstructed labour
		O66.0- O66.9
	O67	Labour and delivery complicated by intrapartum haemorrhage, not elsewhere classified
		O67.0- O67.9
	O68	Labour and delivery complicated by fetal stress
		O68.0- O68.9
	O69	Labour and delivery complicated by umbilical cord complications
		O69.0- O69.9
	O70	Perineal laceration during delivery

		O70.0- O70.9
	O71	Other obstetric trauma
		O71.0- O71.9
	O72	Postpartum haemorrhage
		O72.0- O72.9
	O73	Retained placenta and membranes, without haemorrhage
		O73.0- O73.9
	O74	Complications of anaesthesia during labour and delivery
		O74.0- O74.9
	O75	Other complications of labour and delivery, not elsewhere classified
		O75.0- O75.9
	O81	Single delivery by forceps and vacuum extractor
		O81.3-O81.5
	O82	Single delivery by caesarean section
	O83	Other assisted single delivery
	O90.4	Postpartum acute renal failure
**689**	**Disorder of skin and subcutaneous tissue**	
ICD-9	709	Other disorders of skin and subcutaneous tissue
ICD-10	L00-L08	Infections of the skin and subcutaneous tissue
	L10-L14	Bullous disorders
	L20-L30	Dermatitis and eczema
	L40-L45	Papulosquamous disorders
	L50-L54	Urticaria and erythema
	L55-L59	Radiation-related disorders of the skin and subcutaneous tissue
	L60-L75	Disorders of skin appendages
	L80-L99	Other disorders of the skin and subcutaneous tissue

Table S7. Sex-stratified MR IVW analysis and MR Egger analysis

Outcomes	Men-specific effect									Women-specific effect								
	MR IVW				MR Egger					MR IVW				MR Egger				
	Beta	SE	OR (95%CI)	P *effect*	Beta	SE	OR (95%CI)	P *effect*	P *pleiotropy*	Beta	SE	OR (95%CI)	P *effect*	Beta	SE	OR (95%CI)	P *effect*	P *pleiotropy*
Gout	1.77	0.12	5.85 (4.59, 7.46)	2.22E-15	2.06	0.28	7.81 (4.39, 13.90)	4.87E-08	0.20	1.23	0.30	3.42 (1.84, 6.38)	3.40E-04	1.10	0.47	3.01 (1.16, 7.81)	0.03	0.68
Inflammatory polyarthropathies	0.57	0.07	1.76 (1.53, 2.04)	5.41E-09	0.64	0.13	1.90 (1.45, 2.48)	3.76E-05	0.49	-0.04	0.06	0.96 (0.85, 1.07)	0.43	-0.08	0.07	0.92 (0.79, 1.07)	0.28	0.46
Essential hypertension	0.09	0.04	1.10 (1.02, 1.19)	0.02	-0.07	0.08	0.93 (0.78, 1.10)	0.37	0.02	0.07	0.03	1.08 (1.00, 1.15)	0.04	-0.06	0.07	0.94 (0.82, 1.08)	0.39	0.01
Hypertensive disease	0.09	0.04	1.10 (1.02, 1.19)	0.02	-0.07	0.08	0.93 (0.78, 1.10)	0.37	0.02	0.07	0.03	1.08 (1.00, 1.16)	0.04	-0.06	0.07	0.94 (0.82, 1.08)	0.40	0.00
Myocardial infarction	0.15	0.07	1.17 (1.01, 1.34)	0.03	0.03	0.15	1.03 (0.76, 1.39)	0.84	0.29	0.18	0.11	1.20 (0.96, 1.50)	0.10	0.08	0.15	1.08 (0.79, 1.47)	0.62	0.28
Celiac disease	0.35	0.26	1.42 (0.83, 2.43)	0.19	0.03	0.60	1.03 (0.31, 3.49)	0.96	0.51	0.27	0.16	1.31 (0.94, 1.84)	0.11	0.22	0.28	1.24 (0.70, 2.22)	0.45	0.77
Disorders of metabolism	0.10	0.04	1.10 (1.01, 1.20)	0.04	0.04	0.09	1.04 (0.86, 1.26)	0.70	0.44	0.37	0.38	1.44 (0.66, 3.14)	0.34	0.08	0.75	1.08 (0.24, 4.96)	0.92	0.55
Coronary atherosclerosis	0.05	0.05	1.05 (0.95, 1.16)	0.32	-0.03	0.10	0.97 (0.79, 1.20)	0.79	0.36	0.14	0.06	1.15 (1.02, 1.30)	0.02	0.08	0.10	1.09 (0.88, 1.34)	0.43	0.38
Ischemic heart disease	0.04	0.05	1.04 (0.95, 1.15)	0.39	-0.03	0.10	0.97 (0.79, 1.19)	0.77	0.38	0.14	0.06	1.15 (1.02, 1.30)	0.02	0.07	0.10	1.08 (0.87, 1.33)	0.47	0.33
Angina pectoris	0.03	0.06	1.03 (0.90, 1.17)	0.66	-0.02	0.12	0.98 (0.77, 1.26)	0.89	0.64	0.12	0.08	1.13 (0.97, 1.32)	0.11	0.01	0.12	1.01 (0.80, 1.29)	0.90	0.15
Atrial fibrillation and flutter	-0.03	0.07	0.97 (0.85, 1.11)	0.67	-0.20	0.11	0.81 (0.65, 1.03)	0.08	0.06	0.08	0.08	1.09 (0.92, 1.29)	0.33	-0.02	0.12	0.98 (0.77, 1.25)	0.85	0.18
Circulatory disease	0.05	0.03	1.05 (0.98, 1.12)	0.16	0.01	0.06	1.01 (0.89, 1.13)	0.92	0.39	0.03	0.03	1.03 (0.98, 1.09)	0.23	-0.04	0.05	0.96 (0.86, 1.07)	0.49	0.05
Varicose veins of lower extremity	-0.15	0.11	0.86 (0.69, 1.08)	0.19	-0.15	0.21	0.86 (0.55, 1.33)	0.48	0.97	-0.14	0.07	0.87 (0.76, 0.99)	0.04	-0.14	0.13	0.87 (0.67, 1.13)	0.29	0.98
Disorders of iron metabolism	0.15	0.30	1.16 (0.63, 2.16)	0.62	-0.42	1.05	0.66 (0.08, 5.60)	0.69	0.50	0.37	0.38	1.44 (0.66, 3.14)	0.34	0.08	0.75	1.08 (0.24, 4.96)	0.92	0.55
Hypercholesterolemia	0.22	0.12	1.24 (0.98, 1.57)	0.07	0.20	0.19	1.22 (0.82, 1.81)	0.32	0.91	0.06	0.05	1.06 (0.95, 1.18)	0.29	-0.04	0.12	0.96 (0.75, 1.23)	0.74	0.21
Hypothyroidism	0.20	0.13	1.23 (0.95, 1.59)	0.12	0.21	0.26	1.23 (0.73, 2.08)	0.42	0.98	0.06	0.05	1.06 (0.95, 1.18)	0.29	-0.04	0.12	0.96 (0.75, 1.23)	0.74	0.21
Disorders of thyroid	0.20	0.12	1.22 (0.96, 1.55)	0.09	0.22	0.22	1.25 (0.80, 1.97)	0.32	0.90	0.04	0.05	1.04 (0.94, 1.15)	0.48	-0.04	0.11	0.96 (0.77, 1.20)	0.71	0.28
Benign neoplasm of digestive system	0.06	0.14	1.06 (0.79, 1.41)	0.70	-0.01	0.26	0.99 (0.59, 1.68)	0.97	0.75	-0.11	0.10	0.89 (0.73, 1.10)	0.27	-0.09	0.18	0.91 (0.63, 1.33)	0.63	0.85
Gastritis and duodenitis	-0.04	0.07	0.96 (0.82, 1.11)	0.56	-0.03	0.15	0.97 (0.71, 1.33)	0.83	0.93	-0.03	0.06	0.97 (0.86, 1.09)	0.62	-0.07	0.08	0.93 (0.79, 1.11)	0.41	0.45
Nasal polyps	0.23	0.14	1.25 (0.95, 1.65)	0.11	0.41	0.28	1.51 (0.86, 2.68)	0.15	0.40	-0.23	0.17	0.79 (0.56, 1.11)	0.17	-0.37	0.27	0.69 (0.40, 1.19)	0.17	0.41
Cataract	-0.01	0.08	0.99 (0.84, 1.16)	0.88	-0.13	0.16	0.88 (0.63, 1.23)	0.43	0.38	-0.02	0.06	0.98 (0.86, 1.11)	0.74	-0.10	0.10	0.90 (0.74, 1.10)	0.30	0.20
Poisoning by antibiotics	-0.15	0.23	0.86 (0.53, 1.39)	0.52	0.03	0.45	1.03 (0.41, 2.56)	0.95	0.62	-0.14	0.11	0.87 (0.70, 1.09)	0.21	0.00	0.16	1.00 (0.71, 1.40)	0.99	0.21

Complications of labor and delivery	NA	NA	NA	NA	NA	NA	NA	NA	NA	-0.11	0.07	0.90 (0.78, 1.04)	0.14	-0.18	0.12	0.83 (0.65, 1.06)	0.13	0.31
Other diseases of respiratory system	-0.14	0.11	0.87 (0.69, 1.09)	0.21	-0.35	0.24	0.70 (0.43, 1.14)	0.15	0.28	0.38	0.11	1.46 (1.15, 1.84)	2.62E-03	0.51	0.15	1.66 (1.22, 2.26)	2.27E-03	0.20
Disorder of skin and subcutaneous tissue	0.03	0.05	1.03 (0.93, 1.13)	0.60	0.003	0.09	1.00 (0.84, 1.20)	0.97	0.74	0.003	0.04	1.00 (0.92, 1.09)	0.93	0.04	0.07	1.04 (0.90, 1.20)	0.60	0.46

In sex-stratified MR IVW analysis of 25 disease groups/outcomes (**Supplementary table S6**), 3 disease groups/outcomes (gout, hypertensive disease, and essential hypertension) showed potential causal link with SUA level in both men and women, 3 disease groups/outcomes (inflammatory polyarthropathies, myocardial infarction, and disorders of metabolism) had potential causal link in men, and 4 disease groups/outcomes (coronary atherosclerosis, ischemic heart disease, varicose veins of lower extremity and other diseases of respiratory system) had potential causal link only in women. The sex-stratified MR Egger analysis suggested causal effect of SUA level on gout in both men (OR=7.81, 95%CI: 4.39-13.90, P_{effect}= 4.87e-08, $P_{pleiotropy}$=0.20) and women (OR=3.01, 95%CI: 1.16-7.81, P_{effect}= 0.03, $P_{pleiotropy}$=0.68), on inflammatory polyarthropathies in men (OR=1.90, 95%CI: 1.45-2.48, P_{effect}= 3.76e-05, $P_{pleiotropy}$=0.49), and on other diseases of respiratory system in women (OR=1.66, 95%CI: 1.22-2.26, P_{effect}= 2.27e-03, $P_{pleiotropy}$=0.20).

Table S8. A sensitivity analysis of MR for gout cases defined by multiple criteria.

Case ascertainment	No. of cases*	MR IVW			MR Egger		
		OR (95%CI)	P *effect*	Power	OR (95%CI)	P *effect*	Power
Hospital-diagnosed gout	1,003	4.88 (3.91, 6.09)	3.55E-15	1.00	4.58 (2.72, 7.72)	1.76E-06	1.00
Self-reported gout	1,769	9.78 (8.22, 9.79)	0.00E+00	1.00	14.5 (10.1, 14.9)	2.89E-15	1.00
Hospital-diagnosed or self-reported gout	2,274	8.26 (7.04, 9.60)	0.00E+00	1.00	12.6 (9.06, 17.3)	6.66E-16	1.00

*Note the number of study population is 120,091.

References

1. van der Harst P, Zhang W, Mateo Leach I, et al. Seventy-five genetic loci influencing the human red blood cell. Nature 2012;**492**(7429):369-75.
2. Draisma HH, Pool R, Kobl M, et al. Genome-wide association study identifies novel genetic variants contributing to variation in blood metabolite levels. Nature communications 2015;**6**:7208.
3. Shin SY, Fauman EB, Petersen AK, et al. An atlas of genetic influences on human blood metabolites. Nature genetics 2014;**46**(6):543-50.
4. Kottgen A, Albrecht E, Teumer A, et al. Genome-wide association analyses identify 18 new loci associated with serum urate concentrations. Nature genetics 2013;**45**(2):145-54.
5. Nakayama A, Nakaoka H, Yamamoto K, et al. GWAS of clinically defined gout and subtypes identifies multiple susceptibility loci that include urate transporter genes. Annals of the rheumatic diseases 2017;**76**(5):869-77.
6. Ligthart S, Vaez A, Hsu YH, et al. Bivariate genome-wide association study identifies novel pleiotropic loci for lipids and inflammation. BMC genomics 2016;**17**:443.
7. Kathiresan S, Willer CJ, Peloso GM, et al. Common variants at 30 loci contribute to polygenic dyslipidemia. Nature genetics 2009;**41**(1):56-65.
8. Kamatani Y, Matsuda K, Okada Y, et al. Genome-wide association study of hematological and biochemical traits in a Japanese population. Nature genetics 2010;**42**(3):210-5.
9. Kottgen A, Pattaro C, Boger CA, et al. New loci associated with kidney function and chronic kidney disease. Nature genetics 2010;**42**(5):376-84.
10. Johansen CT, Wang J, Lanktree MB, et al. Excess of rare variants in genes identified by genome-wide association study of hypertriglyceridemia. Nature genetics 2010;**42**(8):684-7.
11. Pattaro C, Teumer A, Gorski M, et al. Genetic associations at 53 loci highlight cell types and biological pathways relevant for kidney function. Nature communications 2016;**7**:10023.
12. Yang Q, Kottgen A, Dehghan A, et al. Multiple genetic loci influence serum urate levels and their relationship with gout and cardiovascular disease risk factors. Circulation Cardiovascular genetics 2010;**3**(6):523-30.
13. Kettunen J, Demirkan A, Wurtz P, et al. Genome-wide study for circulating metabolites identifies 62 loci and reveals novel systemic effects of LPA. Nature communications 2016;**7**:11122.
14. Hunt KA, Zhernakova A, Turner G, et al. Newly identified genetic risk variants for celiac disease related to the immune response. Nature genetics 2008;**40**(4):395-402.
15. Newton-Cheh C, Johnson T, Gateva V, et al. Genome-wide association study identifies eight loci associated with blood pressure. Nature genetics 2009;**41**(6):666-76.
16. Medici M, Porcu E, Pistis G, et al. Identification of novel genetic Loci associated with thyroid peroxidase antibodies and clinical thyroid disease. PLoS genetics 2014;**10**(2):e1004123.
17. Zhernakova A, Stahl EA, Trynka G, et al. Meta-analysis of genome-wide association studies in celiac disease and rheumatoid arthritis identifies fourteen non-HLA shared loci. PLoS genetics 2011;**7**(2):e1002004.
18. Kullo IJ, Shameer K, Jouni H, et al. The ATXN2-SH2B3 locus is associated with peripheral arterial disease: an electronic medical record-based genome-wide association study. Frontiers in genetics 2014;**5**:166.
19. Nikpay M, Goel A, Won HH, et al. A comprehensive 1,000 Genomes-based genome-wide association meta-analysis of coronary artery disease. Nature genetics 2015;**47**(10):1121-30.
20. Liu JZ, van Sommeren S, Huang H, et al. Association analyses identify 38 susceptibility loci for inflammatory bowel disease and highlight shared genetic risk across populations. Nature genetics 2015;**47**(9):979-86.
21. Zemunik T, Boban M, Lauc G, et al. Genome-wide association study of biochemical traits in Korcula Island, Croatia. Croatian medical journal 2009;**50**(1):23-33.
