# Supplementary material for: MR-PheWAS: exploring the causal effect of SUA level on multiple disease outcomes by using genetic instruments in UK Biobank
Source: Ann Rheum Dis. 2018 Feb 6;77(7):1039–47. doi: 10.1136/annrheumdis-2017-212534 (PMC6029646; doi:10.1136/annrheumdis-2017-212534)
Supplement: Supplementary data [file annrheumdis-2017-212534supp002.pdf]

Online Supplementary text

Phenotyping and mapping ICD-10/9 to phecode

ICD-9/10 codes were organized in a hierarchical tree-like structure. The individual ICD-9/10 codes could not be directly used for PheWAS analysis, because they were not designed for representing distinct disease phenotypes. To aggregate the billing codes, the phecode schema has been successfully adopted in a number of PheWAS to combine one or more individual ICD codes into distinct phenotype groups.[1 2] However, since the current version of phecode was developed based on ICD-9-Clinical Modification (CM), the phencode algorithm was not directly applicable to the ICD-10 coding system in the UK Biobank. To develop an aggregation method for PheWAS analysis in UK Biobank, we collaborated with the Electronic Medical Records and Genomics (eMERGE) group of Vanderbilt University Medical Center and mapped ICD-10 codes to phecodes in both direct and indirect ways. We mapped the ICD-10 codes to phecodes directly if their descriptions matched each other regardless of capitalization. Otherwise, we used the unified medical language system (UMLS) to map the ICD-10 code to ICD-9-CM (or map the ICD-10 code to systematized nomenclature of medicine clinical terms [SNOMED CT] code first and then to ICD-9-CM) and then used the previous mapping of ICD-9-CM to phecode to finally link the ICD-10 to phecode. The ICD-9 codes in UK Biobank were directly mapped to the phecodes through the first fourth or full digital codes or through the descriptions regardless of capitalization.

MR IVW, MR Egger and HEIDI test

MR IVW For each independent genetic instrument i, the causal effect of SUA level on the disease outcome (denoted as $b_{xy(i)}$) was estimated by the ratio method, in which the coefficient from the regression of outcome on the genetic variant (using individual-level data from the UK Biobank and denoted as $b_{zy(i)}$) was divided by the coefficient from the regression of SUA level on the genetic variant (using the summary-level GWAS data made available by Kottgen *et al* and denoted as $b_{zx(i)}$).[3] The overall causal effect of SUA level on the outcome mediated by all 31 genetic instruments was estimated by pooling the individual effect estimates of each SNP using the IVW method.[4]

MR Egger Briefly, instead of assuming that the genetic instruments are only associated with SUA level (no pleiotropy criterion of MR), the MR Egger uses a weighted linear regression to regress the effect estimates of SNP-outcome associations against the effect estimates of SNP-SUA associations with the intercept unconstrained. The unconstrained intercept represents the average pleiotropic effects across the genetic variants (with a zero intercept indicating that there are no direct pleiotropic effects or the pleiotropic effects are balanced among the multiple genetic instruments). The slope coefficient from the MR Egger regression represents the overall estimate of the causal effect after accounting for the pleiotropic effects of multiple genetic instruments.[5]

HEIDI test The HEIDI test was firstly proposed by Zhu and colleagues [6], but the principle of this test can

be broadly applied to any pair of traits. The rationale and mathematical theories of this metric are explained elsewhere in detail. [6] The HEIDI method assumes only one causal variant affected both the SUA level and disease outcome (via either vertical pleiotropy [including causality] or horizontal pleiotropy) within a genetic region. If we denote the b_{zx} as the effect estimate of genetic variant on SUA level, and b_{zy} as the effect estimate of genetic variant on disease outcome, the effect estimate of SUA level on disease outcome mediated by genetic component could be calculated by the ratio method:

$$b_{xy} = \frac{b_{zy}}{b_{zx}}$$

If we subscribe the casual variant as SNP_0, under the Hardy-Weinberg equilibrium, for any SNP_i in LD with the causal variant, the effect estimate $b_{xy(i)}$ calculated by the ratio method should be identical to $b_{xy(0)}$:

$$b_{xy(i)} = \frac{b_{zy(i)}}{b_{zx(i)}} = \frac{b_{zy(0)}\, r_{0i} \sqrt{\mathrm{h}_0/h_i}}{b_{zx(0)}\, r_{0i} \sqrt{\mathrm{h}_0/h_i}} = \frac{b_{zy(0)}}{b_{zx(0)}} = b_{xy(0)}$$

where r_{0i} is the LD correlation between the casual variant SNP (0) and SNP (i), and $h_{0/i}$ is determined by the allele frequency ($h = 2p(1-p)$). Thus testing linkage against pleiotropy was equivalent to testing if there was any heterogeneity between $b_{xy(0)}$ and $b_{xy(i)}$. If we defined

$$d_i = b_{xy(i)} - b_{xy(0)}$$

then it was equivalent to testing if $d_i = 0$.

With the matrix of any pair of SNPs(i, j) , the covariance could be calculated by

$$cov\big(b_{xy(i)}, b_{xy(j)}\big) = \frac{r_{ij}}{b_{zx(i)} b_{zx(j)}} \sqrt{var(b_{zy(i)}) var(b_{zy(j)})} + b_{xy(i)} b_{xy(j)} \Big(\frac{r_{ij}}{z_{zx(i)} z_{zx(j)}} - \frac{1}{z_{zx(i)}^2\; z_{zx(j)}^2}\Big)$$

$$cov\big(d_{i,} d_j\big) = cov\big(b_{xy(i)}, b_{xy(j)}\big) - cov\big(b_{xy(i)}, b_{xy(0)}\big) - cov\big(b_{xy(j)}, b_{xy(0)}\big) + var\big(b_{xy(0)}\big)$$

Then, we calculated the Z values of d_i

$$z_{d(i)} = d_i / \sqrt{var(d_i)}$$

Under the null hypothesis, where $d_i = 0$, we have a vector of z_d value that follows the multivariate normal distribution (approximated by the Satterthwaite method) with z_d ~ MVN (0, R), where R is the correlation matrix with the *ij*th element

$$r\big(z_{d(i)}, z_{d(j)}\big) = cov(d_i, d_j) / \sqrt{var(d_i) var(d_j)}$$

The HEIDI statistics was calculated as

$$T_{HEIDI} = \sum_i^m z_{d(i)}^2$$

with m being the number of SNPs associated with SUA level with p<1.57e-03 (equivalent to χ^2 >10).

In the analysis, we defined a region of ±250kb (upstream and downstream) around the locus associated with SUA level. For each locus, we calculated the HEIDI statistic only including SNPs that were associated with SUA level at p<1.57e-03 (equivalent to χ^2 >10) in order to avoid very weak instruments and to increase the power. The larger the heterogeneity, the smaller the HEIDI's P-value, and the higher the probability of association is caused by LD. The pattern of regional genetic association with SUA level and disease outcome was visualized by the LocusZoom.[7]

Reference

1. Denny JC, Ritchie MD, Basford MA, et al. PheWAS: demonstrating the feasibility of a phenome-wide scan to discover gene-disease associations. Bioinformatics (Oxford, England) 2010;**26**(9):1205-10.
2. Denny JC, Bastarache L, Ritchie MD, et al. Systematic comparison of phenome-wide association study of electronic medical record data and genome-wide association study data. Nature biotechnology 2013;**31**(12):1102-10.
3. Kottgen A, Albrecht E, Teumer A, et al. Genome-wide association analyses identify 18 new loci associated with serum urate concentrations. Nature genetics 2013;**45**(2):145-54.
4. Burgess S, Butterworth A, Thompson SG. Mendelian randomization analysis with multiple genetic variants using summarized data. Genetic epidemiology 2013;**37**(7):658-65.
5. Bowden J, Davey Smith G, Burgess S. Mendelian randomization with invalid instruments: effect estimation and bias detection through Egger regression. International journal of epidemiology 2015;**44**(2):512-25.
6. Zhu Z, Zhang F, Hu H, et al. Integration of summary data from GWAS and eQTL studies predicts complex trait gene targets. Nature genetics 2016;**48**(5):481-7.
7. Pruim RJ, Welch RP, Sanna S, et al. LocusZoom: regional visualization of genome-wide association scan results. Bioinformatics (Oxford, England) 2010;**26**(18):2336-7.
