# Supplementary figures and images for: MR-PheWAS: exploring the causal effect of SUA level on multiple disease outcomes by using genetic instruments in UK Biobank
Source: Ann Rheum Dis. 2018 Feb 6;77(7):1039–47. doi: 10.1136/annrheumdis-2017-212534 (PMC6029646; doi:10.1136/annrheumdis-2017-212534)

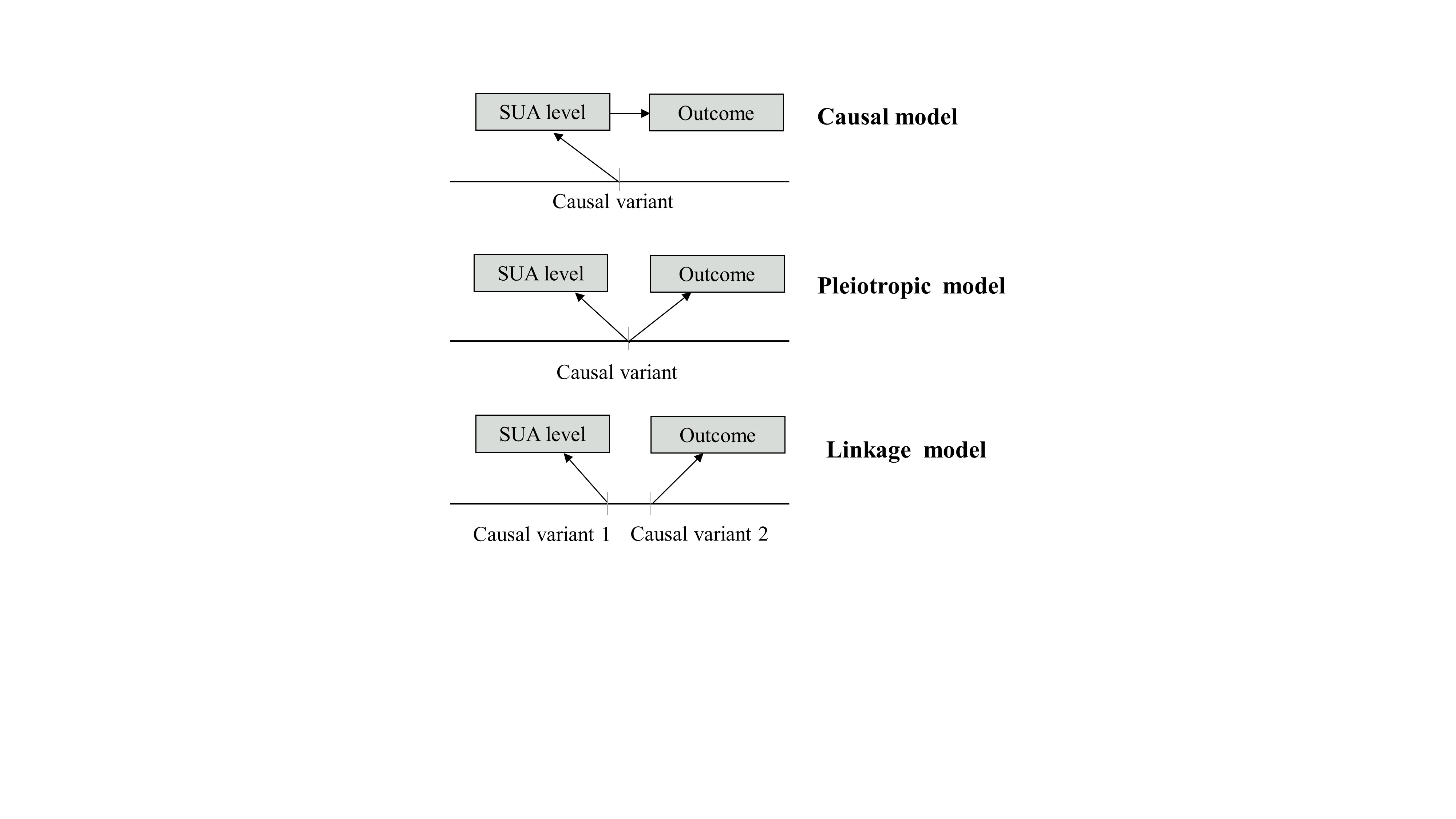

Supplement: Supplementary data [file annrheumdis-2017-212534supp003.jpg]

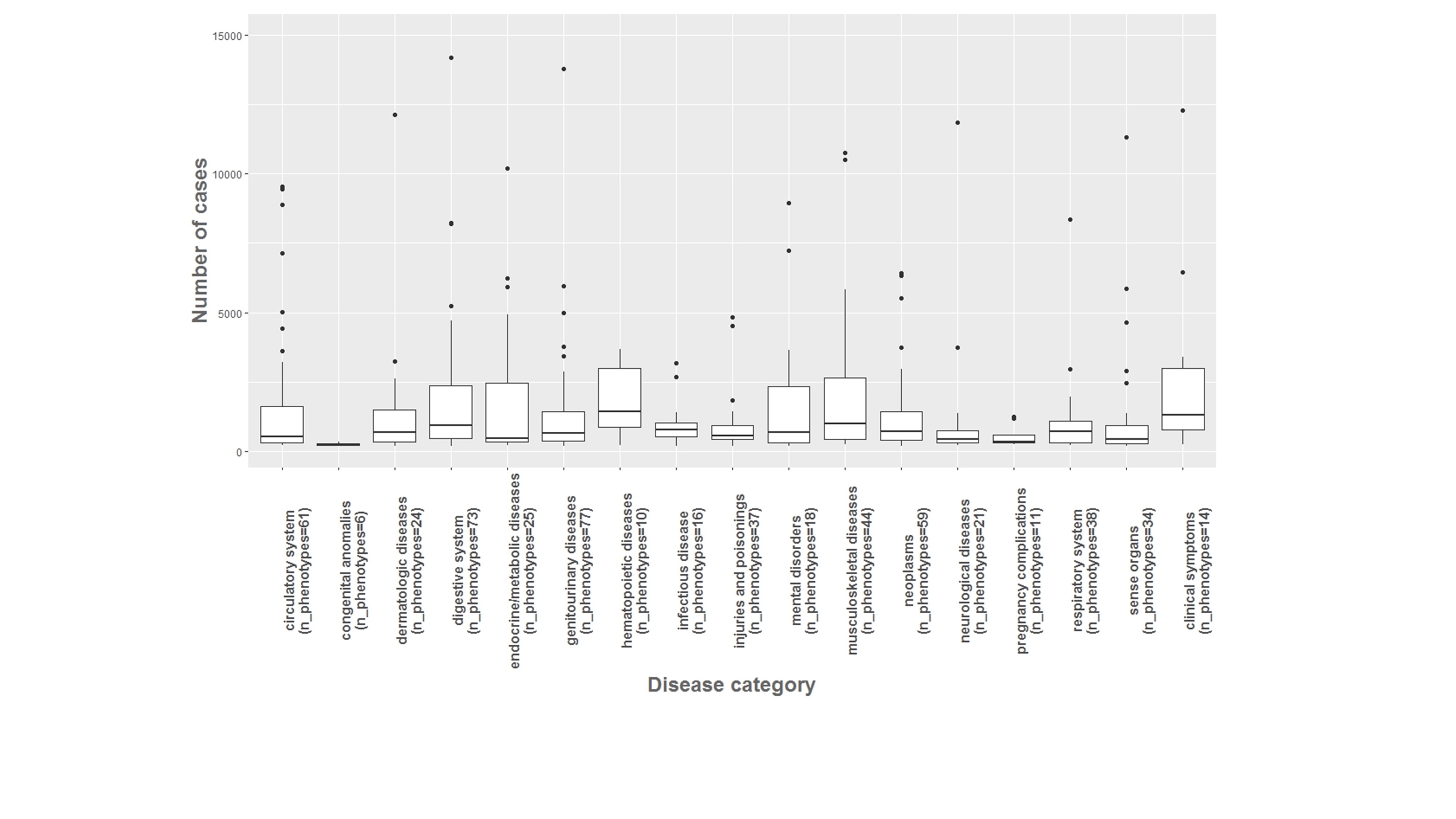

Supplement: Supplementary data [file annrheumdis-2017-212534supp004.jpg]

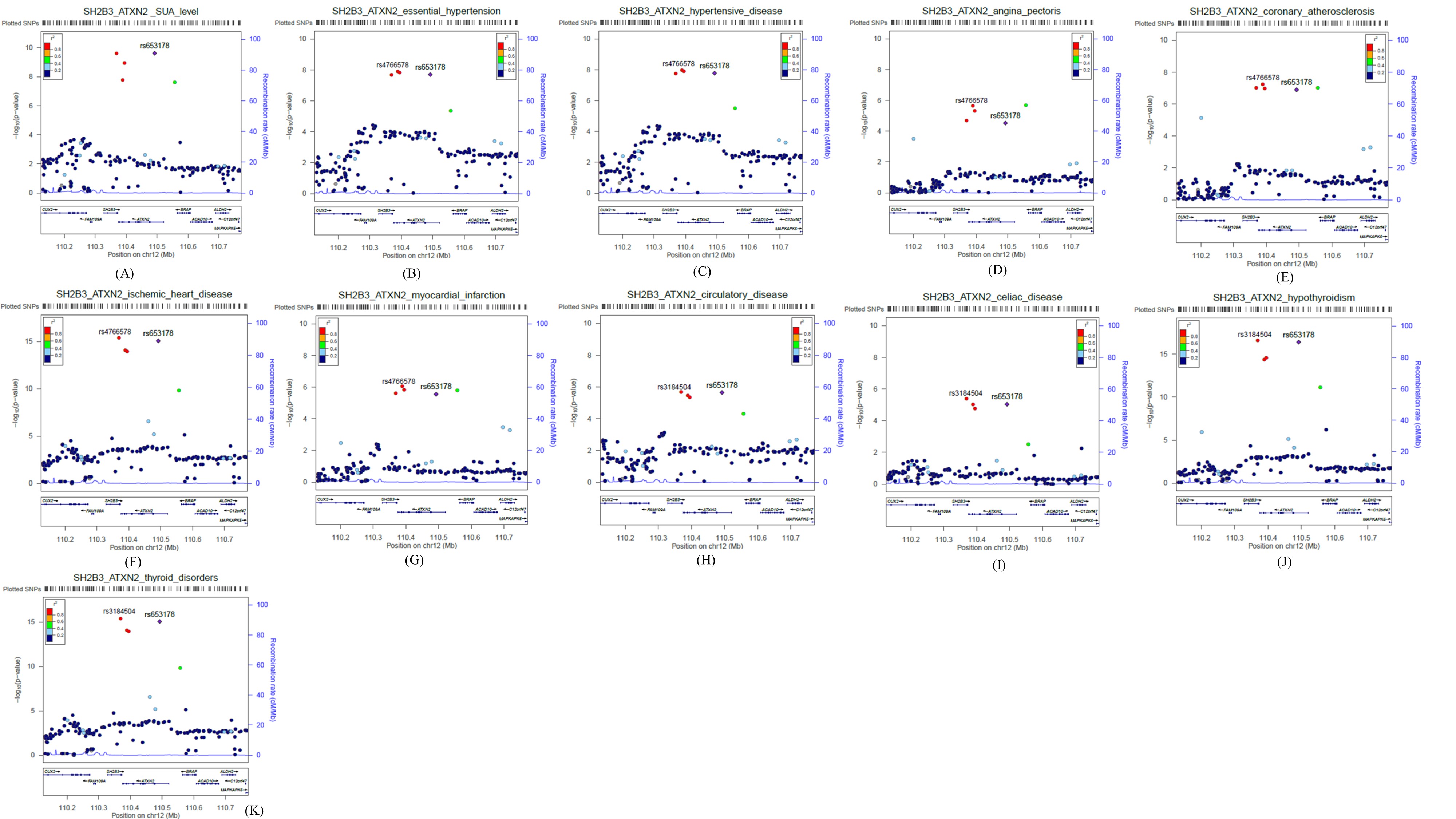

Supplement: Supplementary data [file annrheumdis-2017-212534supp012.jpg]

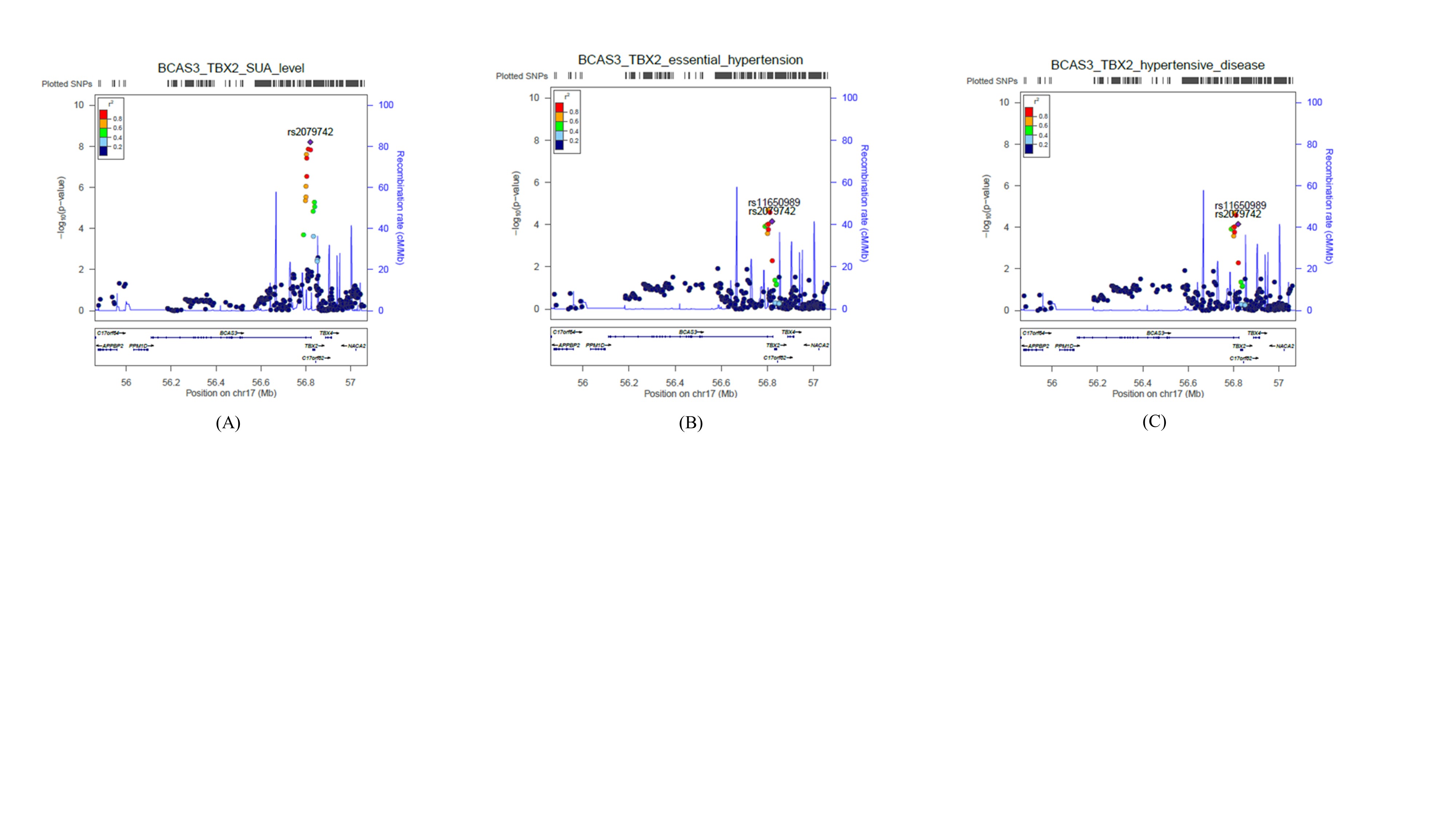

Supplement: Supplementary data [file annrheumdis-2017-212534supp013.jpg]

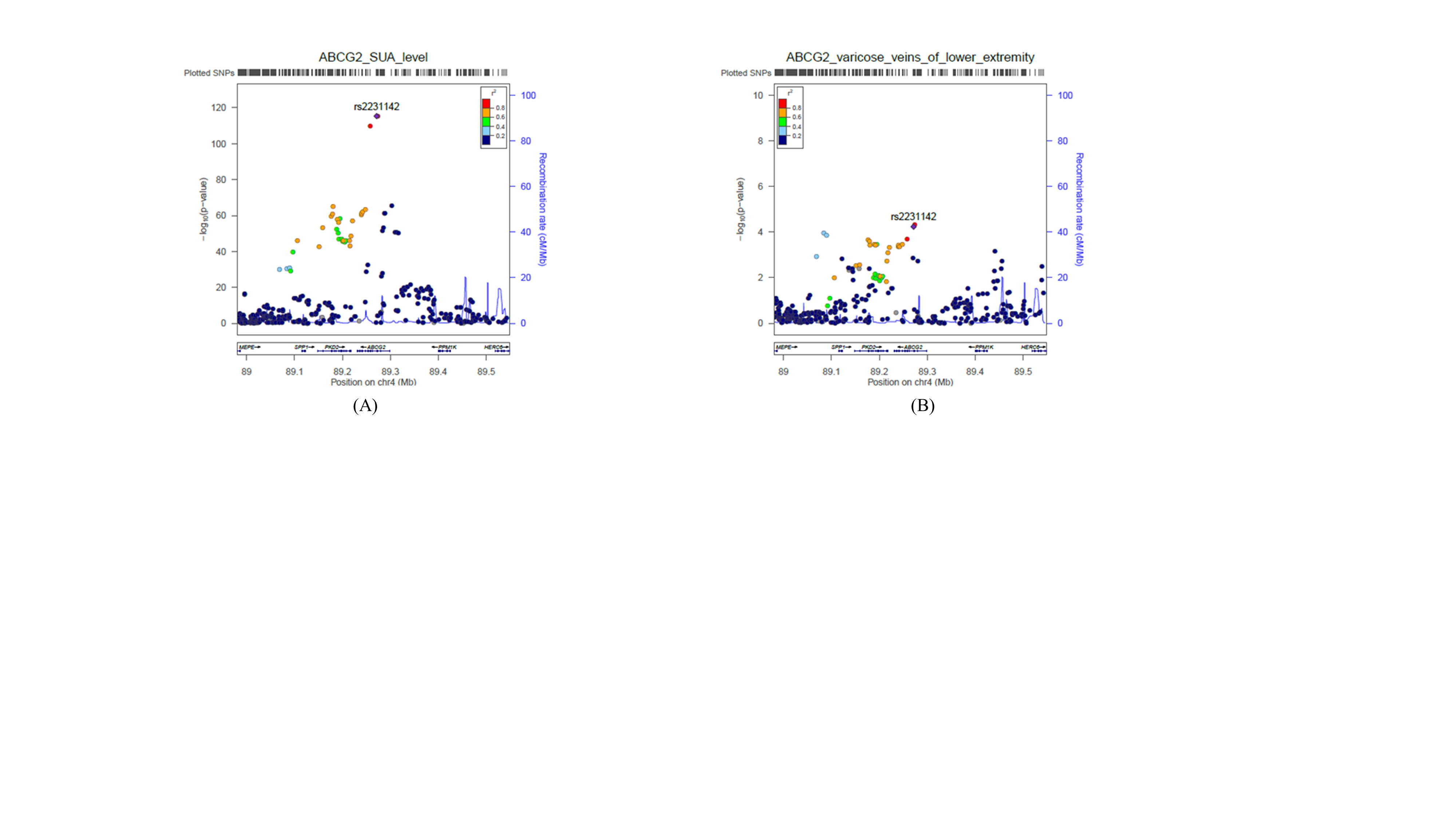

Supplement: Supplementary data [file annrheumdis-2017-212534supp014.jpg]

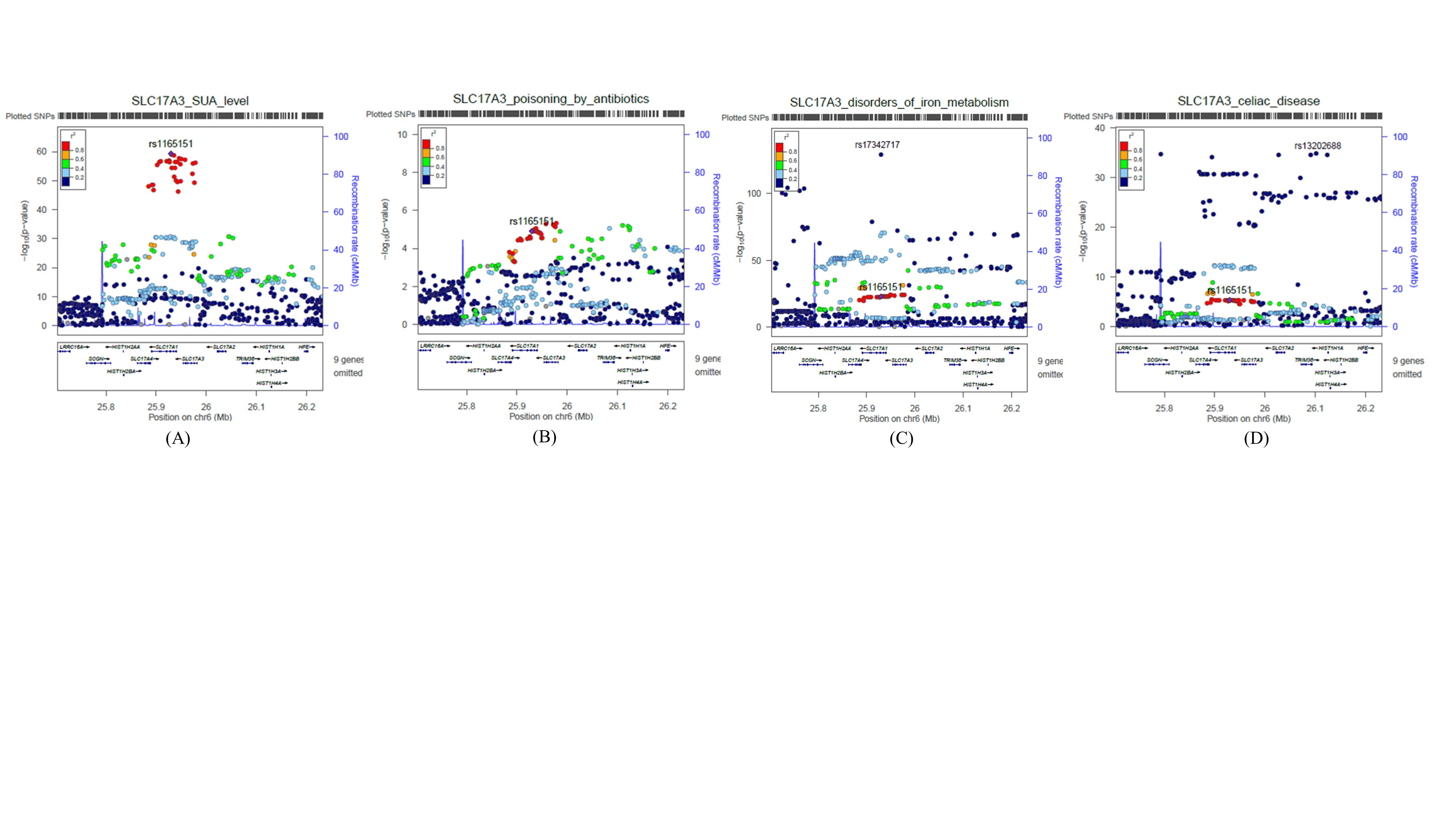

Supplement: Supplementary data [file annrheumdis-2017-212534supp015.jpg]

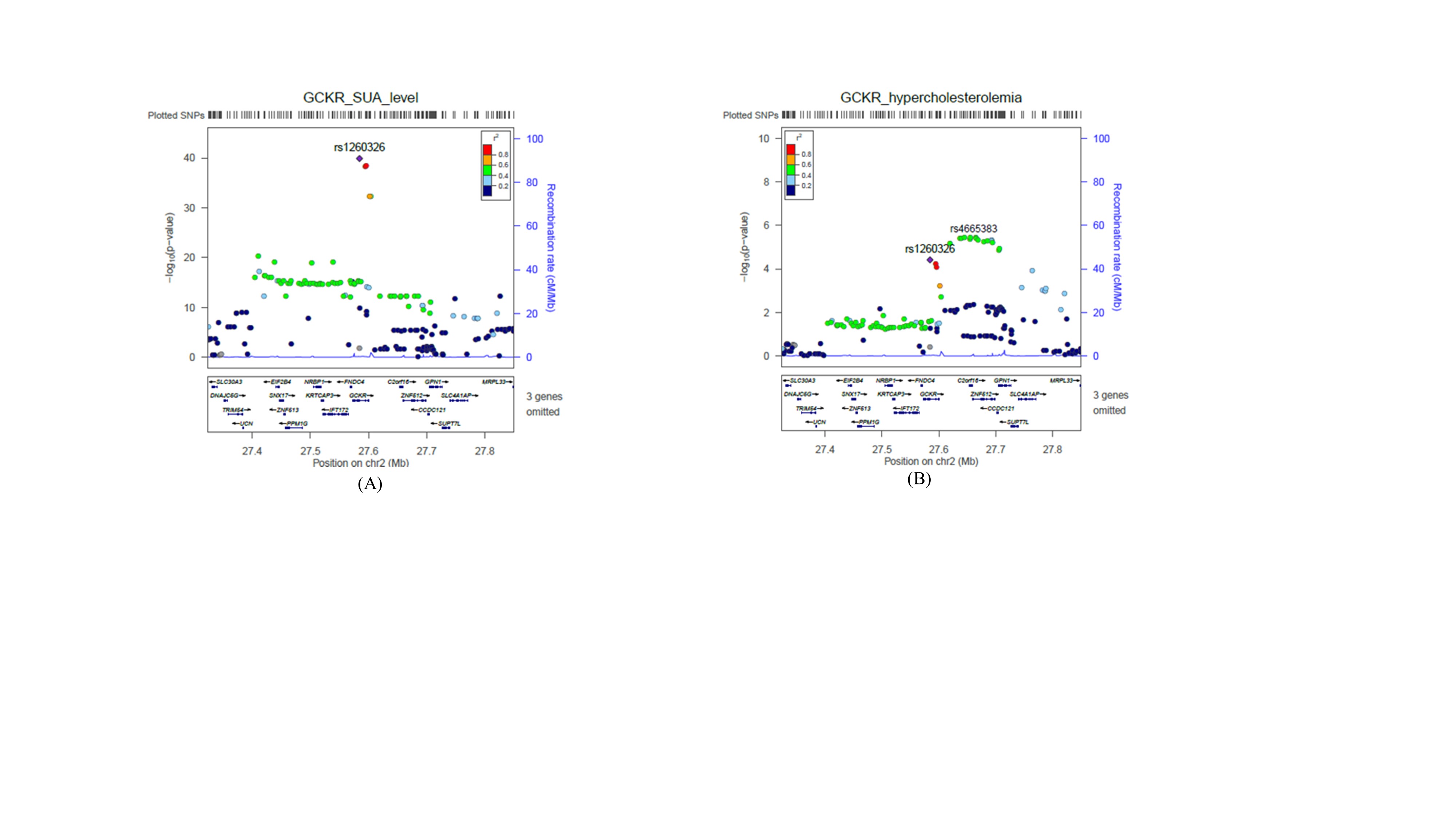

Supplement: Supplementary data [file annrheumdis-2017-212534supp016.jpg]

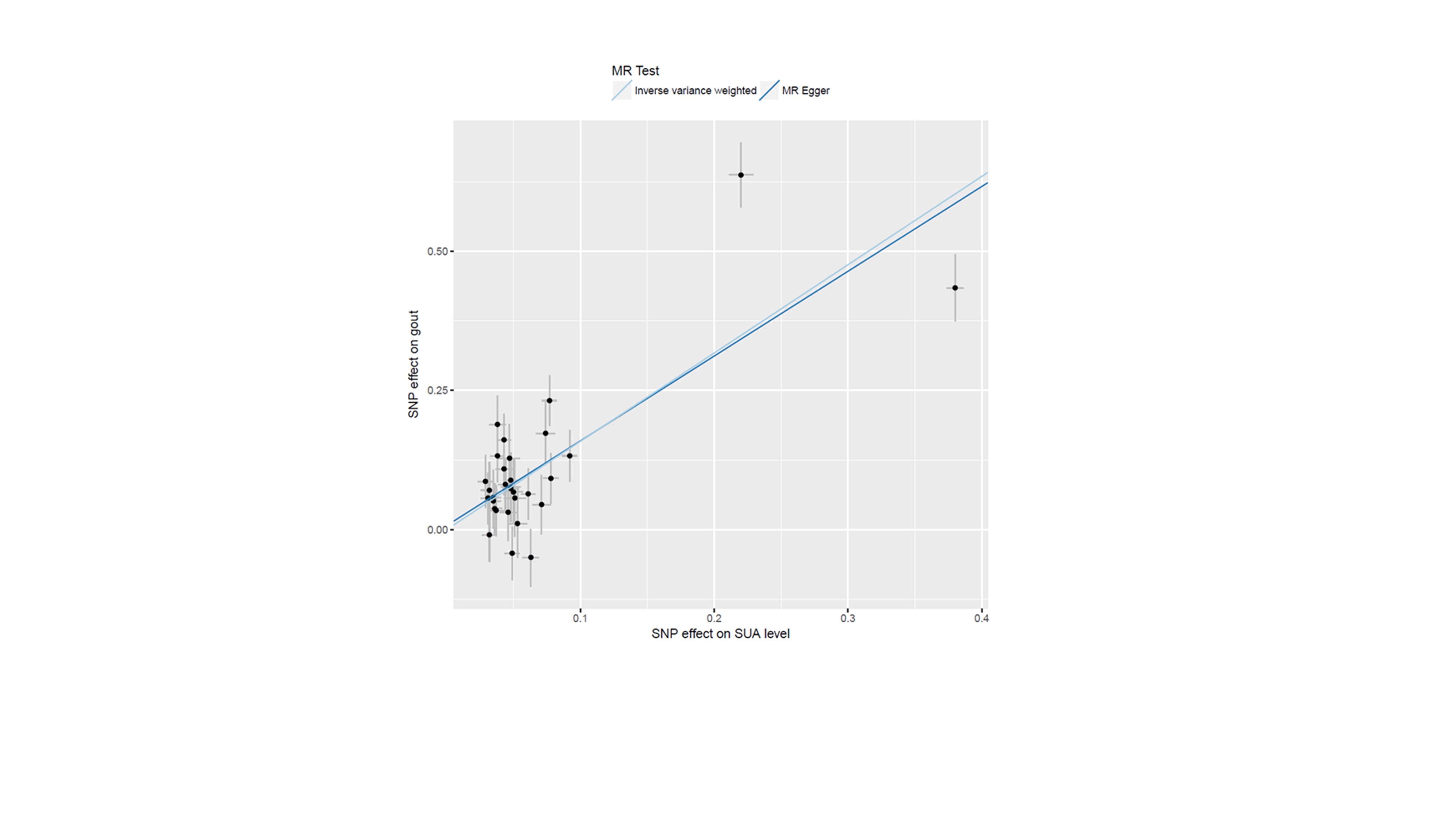

Supplement: Supplementary data [file annrheumdis-2017-212534supp005.jpg]

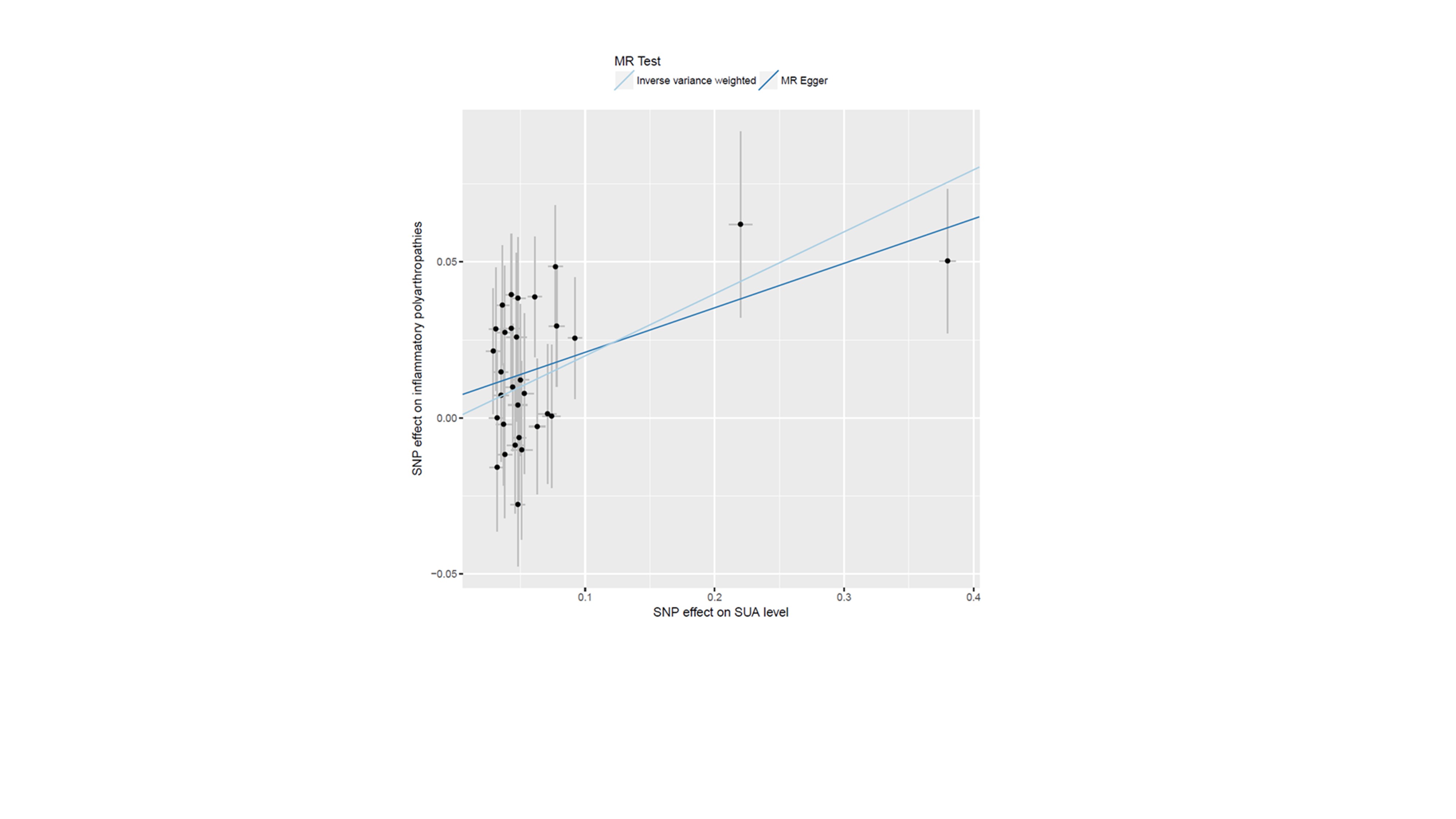

Supplement: Supplementary data [file annrheumdis-2017-212534supp006.jpg]

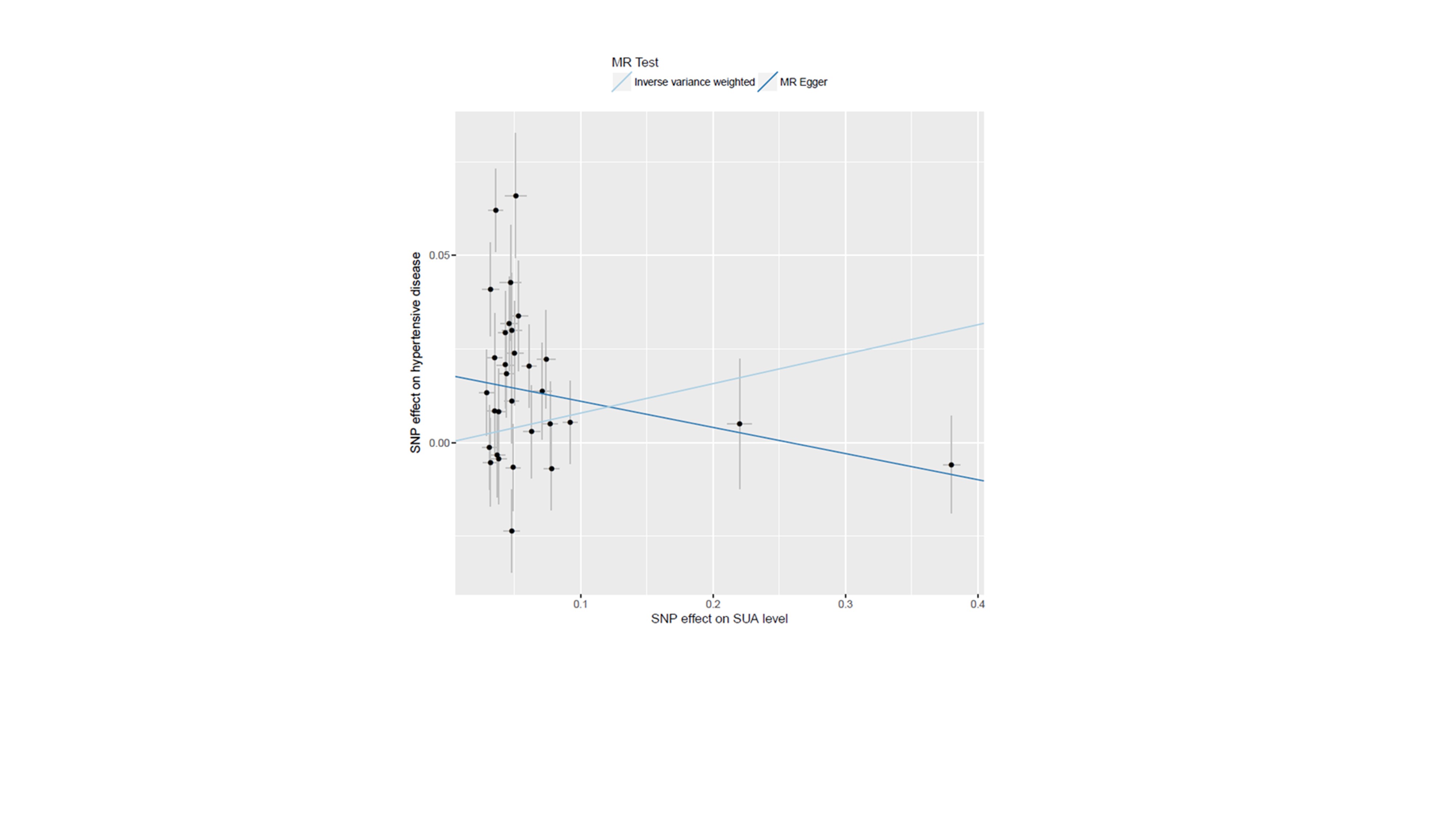

Supplement: Supplementary data [file annrheumdis-2017-212534supp007.jpg]

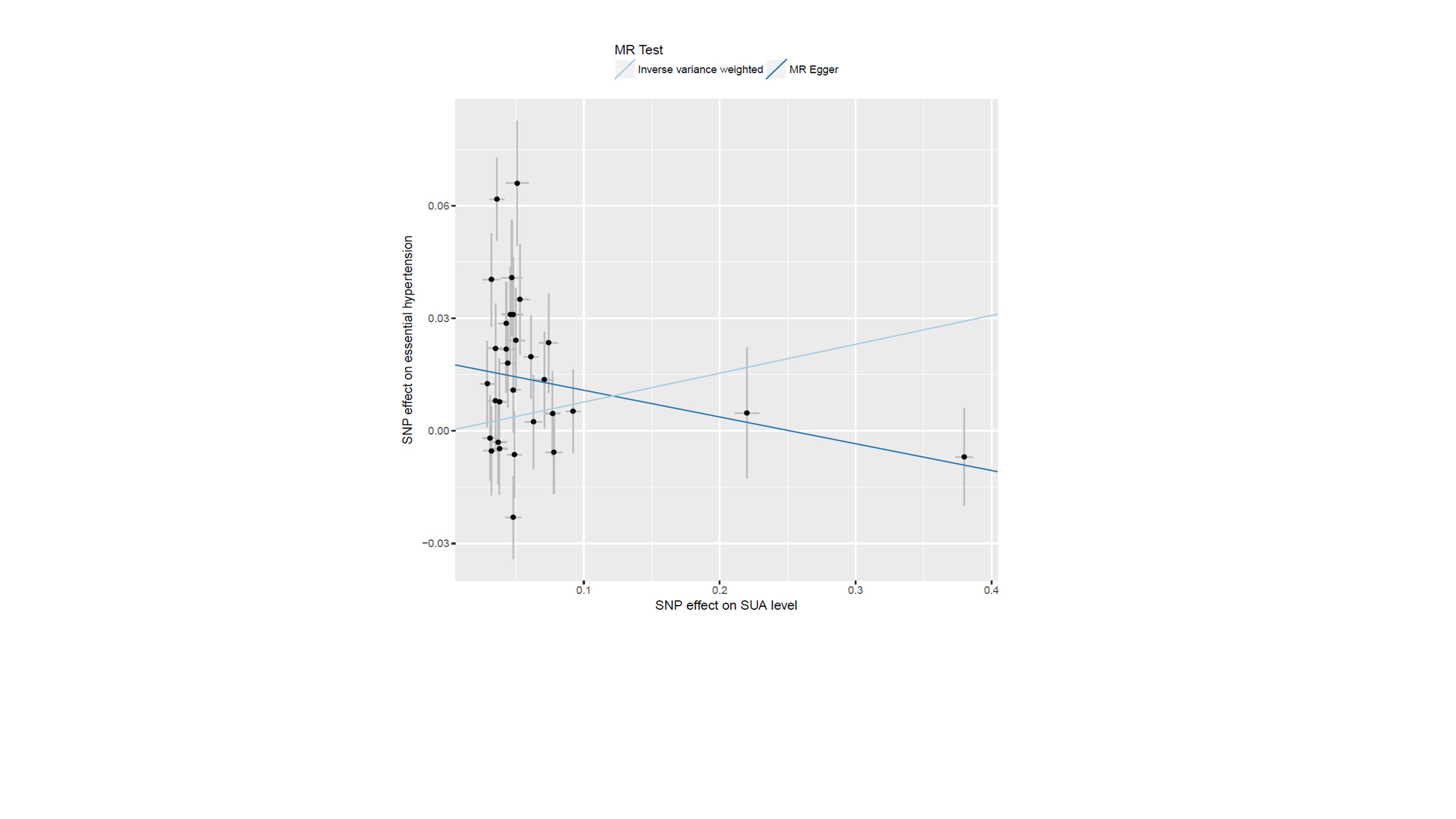

Supplement: Supplementary data [file annrheumdis-2017-212534supp008.jpg]

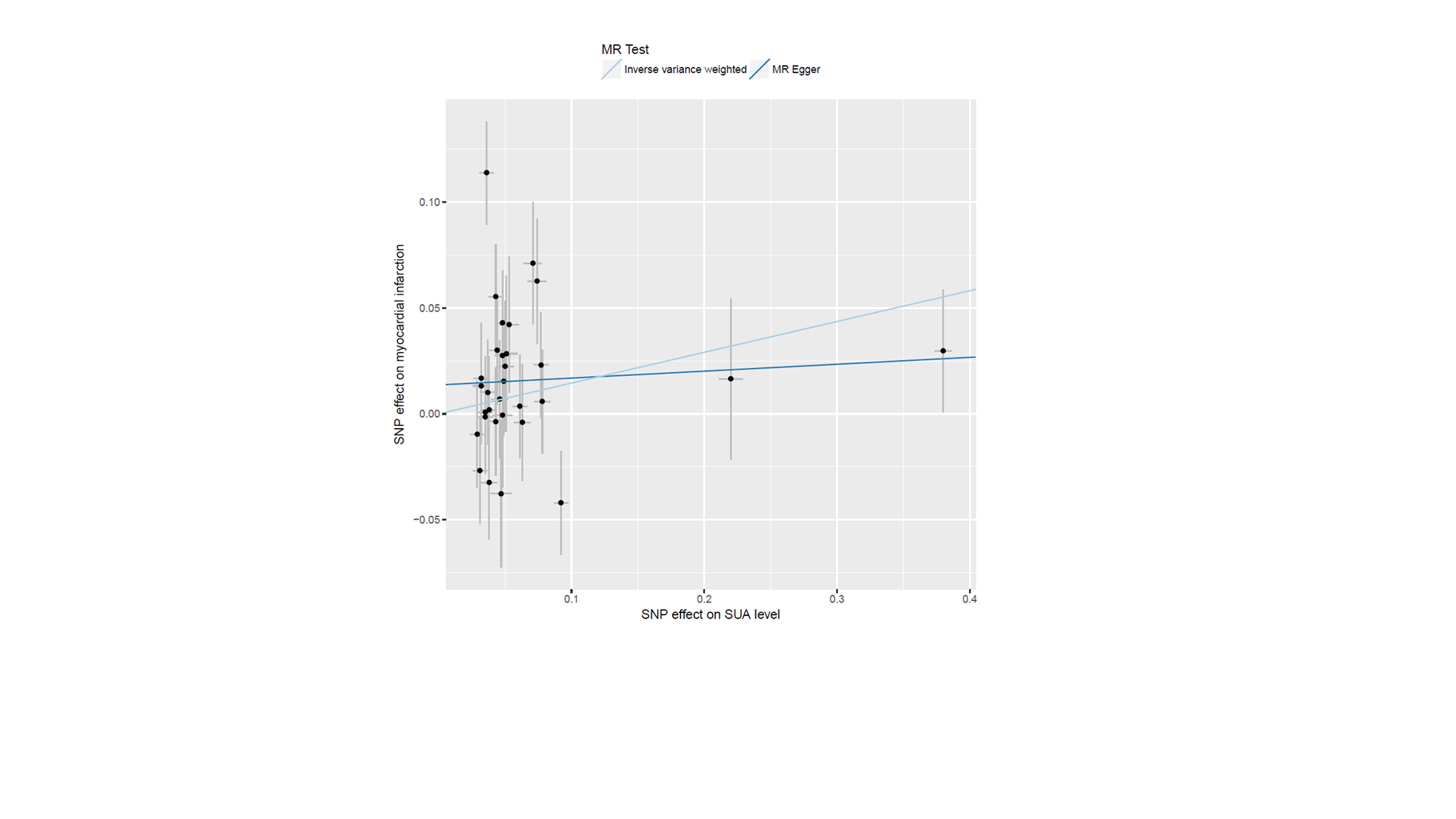

Supplement: Supplementary data [file annrheumdis-2017-212534supp009.jpg]

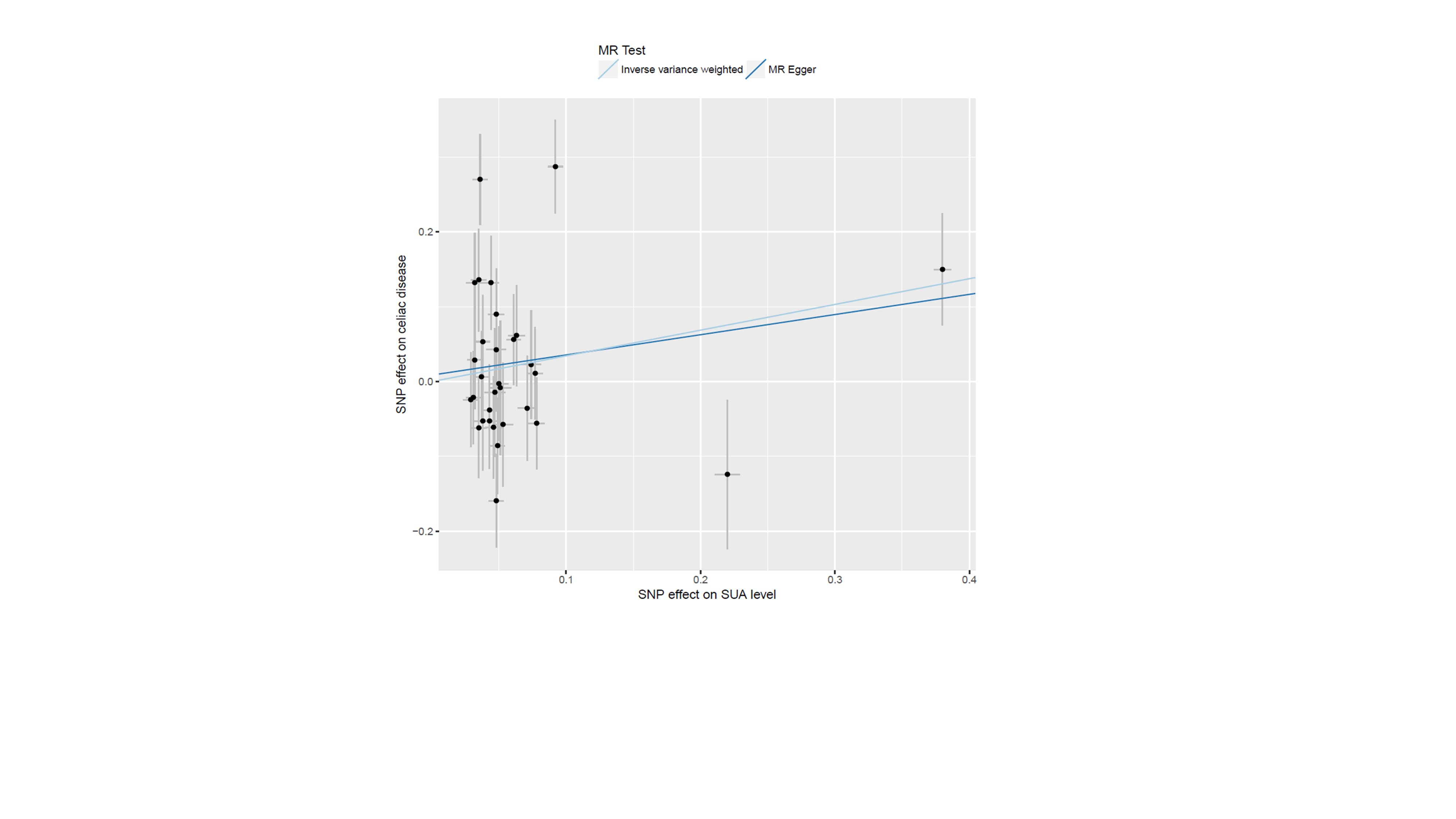

Supplement: Supplementary data [file annrheumdis-2017-212534supp010.jpg]

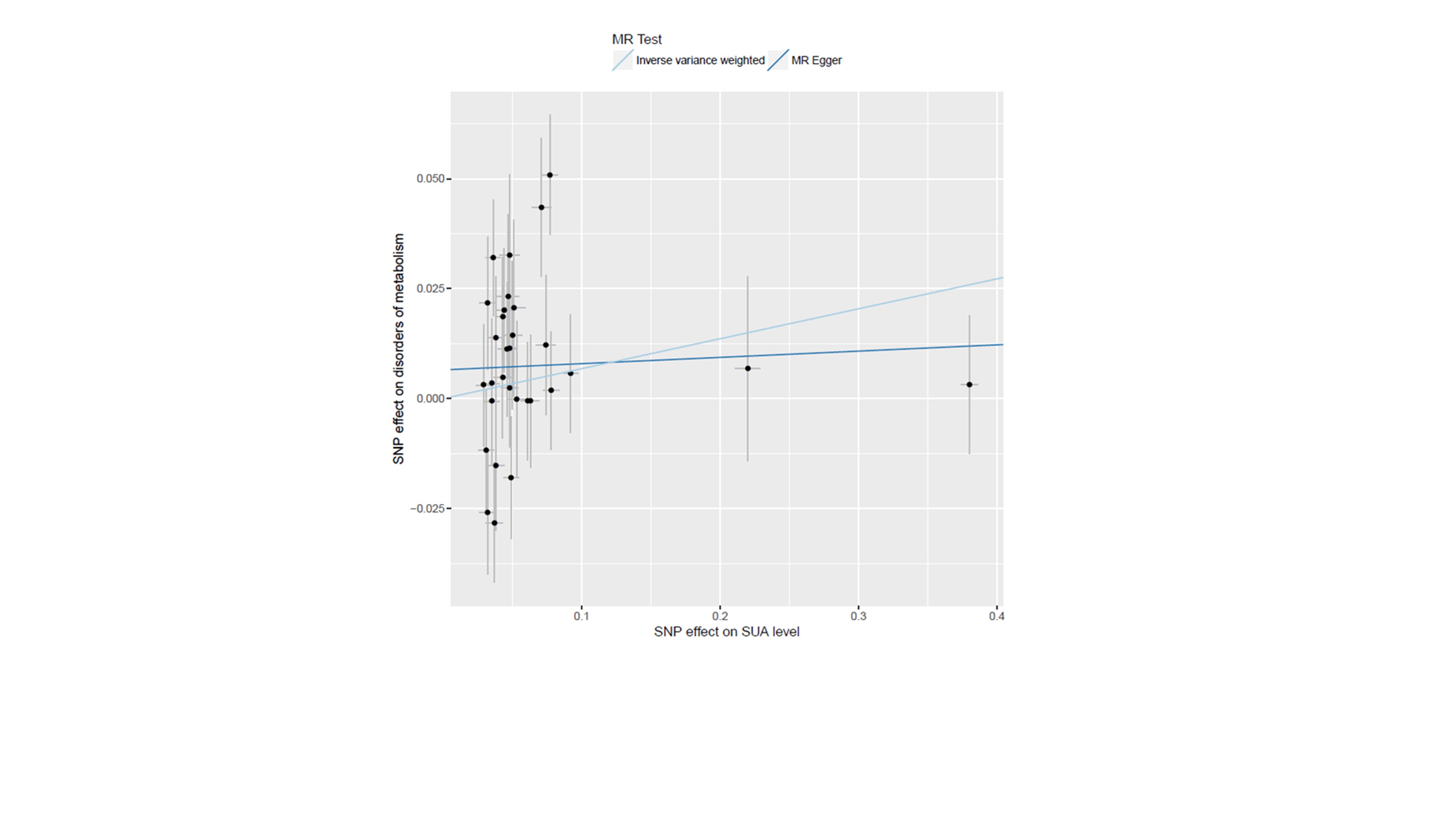

Supplement: Supplementary data [file annrheumdis-2017-212534supp011.jpg]
